# Visualization of Tetrahedral Li in the Alkali Layers of Li-Rich Layered Metal Oxides

**DOI:** 10.1021/jacs.4c05556

**Published:** 2024-08-14

**Authors:** Weixin Song, Miguel A. Pérez-Osorio, Jun Chen, Zhiyuan Ding, John-Joseph Marie, Mikkel Juelsholt, Robert A. House, Peter G. Bruce, Peter D. Nellist

**Affiliations:** † Department of Materials, University of Oxford, Oxford OX1 3PH, U.K.; ‡ The Faraday Institution, Didcot OX11 0RA, U.K.; § The Henry Royce Institute, Oxford OX1 3PH, U.K.

## Abstract

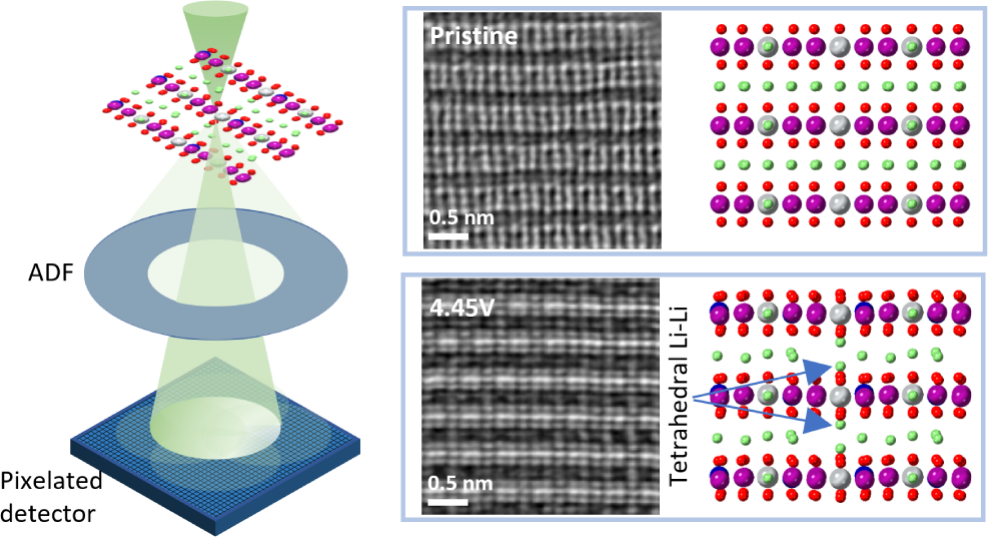

Understanding Li^+^ ion diffusion pathways in Li-rich layered transition metal (TM) oxides is crucial for understanding the sluggish kinetics in anionic O^2–^ redox. Although Li diffusion within the alkali layers undergoes a low-barrier octahedral–tetrahedral–octahedral pathway, it is less clear how Li diffuses in and out of the TM layers, particularly given the complex structural rearrangements that take place during the oxidation of O^2–^. Here, we develop simultaneous electron ptychography and annular dark field imaging methods to unlock the Li migration pathways in Li_1.2_Ni_0.13_Mn_0.54_Co_0.13_O_2_ associated with structural changes in the charge–discharge cycle. At the end of TM oxidation and before the high-voltage O oxidation plateau, we show that the Li migrating out of the TM layers occupies the alkali-layer tetrahedral sites on opposite sides of the TM layers, forming Li–Li dumbbell configurations, consistent with the density functional theory calculations. Also occurring are the TM migration and phase transition from O3 to O1 stacking, leading to unstable tetrahedral Li and the absence of Li contrast in imaging. Upon further Li deintercalation to 4.8 V, most of the tetrahedral Li are removed. After discharging to 2 V, we did not identify the reformation of tetrahedral Li but observed permanently migrated TMs at the alkali-layer sites, disfavoring the Li occupying the tetrahedral sites for diffusion. Our findings suggest a landscape of Li diffusion pathways in Li-rich layered oxides and strategies for minimizing the disruption of Li diffusion.

## Introduction

Layered transition metal (TM) oxides are important cathode materials for secondary-ion batteries because of their combined high energy and power densities,^1^ which have contributed significantly to the success of Li-ion batteries in portable electronic devices and electric vehicles. The ease of Li-ion diffusion through the battery components determines the battery’s rate performance^2,3^ and diffusion through the cathode can be a limiting factor. Understanding the pathways and the barriers to Li^+^ ion diffusion in layered oxides is paramount to inform the structure and diffusion paths for high Li mobility, such as the optimization of TM-layered compositions.^4^ Li diffusion in the alkali layers of a stoichiometric metal oxide has been predicted by first-principles calculations to be a hopping process from the octahedral to octahedral sites through face-sharing intermediate tetrahedral sites.^5^ This octahedral–tetrahedral–octahedral (*o*–*t*–*o*) route has a lower activation energy than the direct jump between the octahedral sites across the shared edges;^5^ however, the activation barrier is strongly affected by the size of the tetrahedral sites and the electrostatic interaction between the tetrahedral-site Li and the octahedral-site cations in TM layers that share a face with the tetrahedron.^2,3^

In conventional layered oxides, Li intercalation and deintercalation are charge-compensated by the reduction and oxidation (redox) of the TM ions.^6^ Li-rich layered oxides, such as Li_1.2_Ni_0.13_Mn_0.54_Co_0.13_O_2_, open avenues to increase the specific capacities by going beyond TM-redox and activating additional anionic O^2–^ redox. Li_1.2_Ni_0.13_Mn_0.54_Co_0.13_O_2_ can deliver a high reversible capacity of over 250 mAh g^–1^ in cycling.^7^ In Li-rich oxides, Li exists not only in the alkali layers but also in the TM layers, resulting in Li–O–Li configurations. Such structures can produce O 2p nonbonding states, which are understood to allow the O-redox reaction.^8^ Since the discovery of O-redox in the 2000s,^9,10^ most research has focused on understanding the O-redox mechanism and associated degradation issues in energy storage. Debates continue on the processes accompanying the high voltage plateau after TM-oxidation. Several theories have been proposed for the nature of O oxidation: formation of electron holes pinned in the O 2p orbitals,^11^ O_2_^2–^ and peroxo-like O–O dimers (O_2_^*n*–^)^12,13^ and short TM=O bonds.^14^ Recently, evidence has been found identifying the formation of O_2_ molecules trapped in TM vacancies.^15^ Regardless of the precise mechanism, the kinetics of the O-redox reaction are typically sluggish,^7,16^ manifesting in more pronounced voltage hysteresis at high rates, which has recently been related to a slow ligand-to-metal charge transfer process.^17^ Here, we examine the lithium diffusion in Li-rich materials, which accompanies the electron transfer and may play an important role in explaining the electrochemical behavior.

The Li-migration mechanism of Li-rich oxides was studied by first-principles calculations and nuclear magnetic resonance (NMR).^15,18,19^ The studies suggest that at an early charging stage of Li-rich oxides, the Li ions in the alkali layers are the first to deintercalate, allowing a trivacancy to be formed in the alkali layers.^18,19^ Such a trivacancy is formed in three octahedral sites that are edge-sharing with the Li-containing octahedra in the TM layers. Once the trivacancies are generated, Li in the alkali layer migrates from its octahedral site into the tetrahedral site that is face-sharing with the Li-containing octahedra in the TM layers.^19^ This Li of the TM layers will then migrate into the opposite tetrahedral site relative to the TM layer at the same time to reduce the total energy of the structure and result in a Li–Li dumbbell across a Li vacancy in the TM layers, which can occur at the beginning of the plateau (BOP, ∼4.45 V).^18,19^ The Li–Li dumbbells with Li at the tetrahedral sites of the alkali layers are predicted to be stable in the structure and require a higher voltage to be removed on charging beyond the BOP state.^19^ However, the theoretically calculated voltages for extracting the tetrahedral Li are inconsistent in the literature.^18,19^ In addition, the Li-diffusion mechanism and mobility can be altered by the TM migration and cation disorder,^20^ which are typical structural changes in Li-rich oxides during the electrochemical cycles.^21^ The Li mobility is not always sluggish in the O redox and can be rather fast, which is reported in Li-rich oxyfluoride with partial TM disorder.^1^ As yet, the understanding of Li diffusion in the Li-rich oxides is still inadequate. NMR studies suggest Li migration from the alkali layers or TM layers but cannot map the Li diffusion pathways over different crystalline sites because of the lack of spatial resolution.^15,18^ High spatial-resolution imaging of Li using electron microscopy has never been achieved because of the challenges in imaging Li.

In this work, we performed focused-probe electron ptychography with synchronized annular dark field (ADF) imaging on Li_1.2_Ni_0.13_Mn_0.54_Co_0.13_O_2_ to unlock the visualization of Li in charge–discharge cycling and the causes of blocking of Li migration. By the quantitative analysis of the phase image reconstructed by the Wigner distribution deconvolution (WDD) algorithm, we demonstrate the formation of tetrahedral Li–Li dumbbells in Li_1.2_Ni_0.13_Mn_0.54_Co_0.13_O_2_ at the BOP state of charge. In combination with density functional theory (DFT) calculations, we show that a Li–Li dumbbell is formed by two Li at opposite tetrahedral sites of the alkali layers across a TM-layer vacancy. The tetrahedral Li–Li dumbbells are removed by charging to a cutoff of 4.8 V, consistent with our theoretical calculations on the voltages required for extracting tetrahedral Li. In addition, the tetrahedral Li–Li dumbbells are found to be stable in the O3 phase of BOP Li_1.2_Ni_0.13_Mn_0.54_Co_0.13_O_2_ and are associated with the high-voltage plateau. In contrast, the dumbbells are absent in the O1 phase changed from the O3 phase, and also not seen in the structures with in-plane and out-of-plane TM migration occurring during charging. The structural evolutions of Li_1.2_Ni_0.13_Mn_0.54_Co_0.13_O_2_ can, therefore, make the tetrahedral sites unstable for Li. If the energy associated with tetrahedral site occupancy is sufficiently high, then it could block Li diffusion resulting in capacity fading. If there are Li vacancies in the TM layers, they are suggested to promote the in-plane TM migration during cycling and lead to voltage fading.^15,18^

## Results and Discussion

### Atomic Structure of Li_1.2_Ni_0.13_Mn_0.54_Co_0.13_O_2_

**Figure 1:**
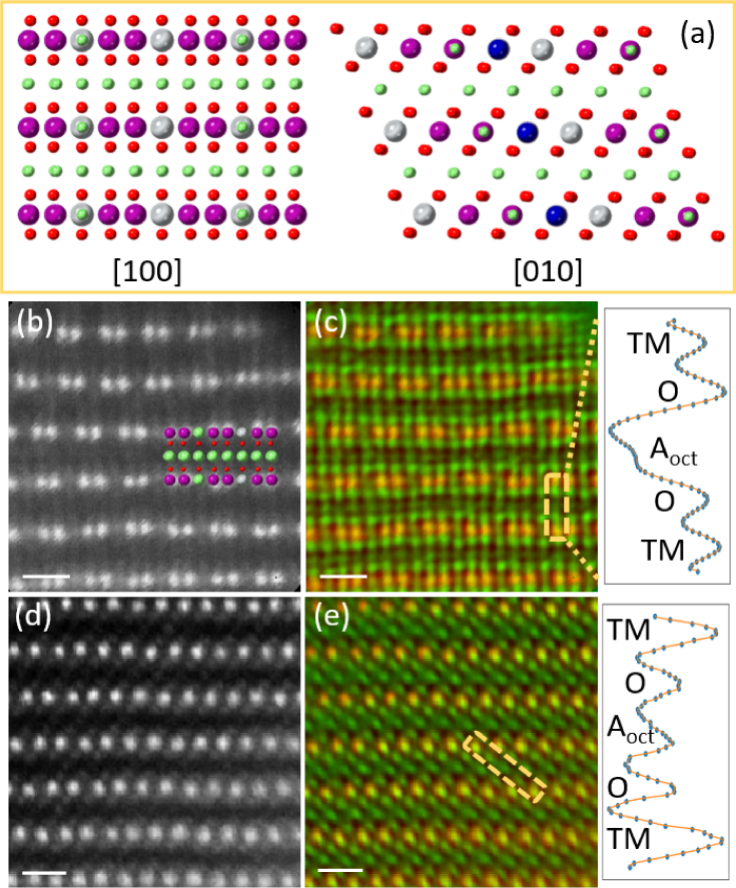
Ptychographic imaging of pristine Li_1.2_Ni_0.13_Mn_0.54_Co_0.13_O_2_. (a) Schematic representation of the DFT-optimized structure in the pristine state along the [100] and [010] zone axes, respectively. The purple spheres are Mn, blue Co, gray Ni, red O, and green Li. Focused probe electron ptychography enables aberration-corrected ADF and ptychographic images to be acquired simultaneously. Imaging is performed along the (b,c) mixed zone axes of [100], [110], and [110], and (d,e) [010]. To guide the visualization, the phase image is colored in green, and the atom positions showing the ADF contrast are colored in orange. (b,d) ADF image. (c,e) Colored ptychographic phase image. Scan distortion and sample drift may occur in data acquisition because of the speed of the pixelated detector. The line profiles in (c) and (e) are obtained from the square region highlighted in orange. A_oct_ indicates the octahedral sites of the alkali layers. The scale bar of all of the images is 0.5 nm.

Figure 1a schematically shows the layered structure of the as-prepared Li_1.2_Ni_0.13_Mn_0.54_Co_0.13_O_2_ along the [100] and [010] zone axes, respectively. The cations in the TM layers are arranged into a honeycomb-like ordered structure between Li/Ni and Mn/Co in a monoclinic structure (space group: *C*2/*m*) though there are still debates over whether Li_1.2_Ni_0.13_Mn_0.54_Co_0.13_O_2_ is a binary-phase composite or single phase.^15^ The O lattice is known as O3 stacking (ABCABC type), which is similar to a face-centered cubic (fcc) lattice accommodating the cations at the octahedral sites. Conventional ADF imaging of Li_1.2_Ni_0.13_Mn_0.54_Co_0.13_O_2_, see Figure S1, is sensitive only to the heavy TMs because the contrast from TMs overwhelms that from the light Li and O atoms. To visualize the light atoms, we performed 4D scanning transmission electron microscopy (STEM) electron ptychography and reconstructed the phase images from the 4D STEM data sets, which is sensitive to the light atoms.^21,22^ Focused-probe electron ptychography enables recording of the aberration-corrected ADF image simultaneously, and all atomic species can be identified.

The ADF image in Figure S1 shows that the TM layers of Li_1.2_Ni_0.13_Mn_0.54_Co_0.13_O_2_ stack with rotational stacking faults along the *c* direction, akin to the projection of crystal domains along different zone axes of [100], [110], and [10], respectively. Consequently, a single image contains views along multiple crystal orientations. We refer to this projection direction as a mixed projection along the [100], [110], and [10] zone axes, which, however, does not imply any physical mixing of atomic species. Along the mixed zone axis, Figure 1b shows an ADF image of pristine Li_1.2_Ni_0.13_Mn_0.54_Co_0.13_O_2_. Figure S2 shows a simultaneous ptychographic image. To guide the visualization, we generate a colored ptychographic image by combining the simultaneous ADF and ptychographic images, with the phase contrast in green and the ADF contrast in orange. Figure 1c shows the colored ptychographic image where the TMs and Li and O atoms are directly distinguished. The line profile along the marked region, which is between two adjacent TM layers in Figure 1c, indicates that the Li ions of the alkali layers occupy the octahedral sites. The images along the [010] zone axis in Figure 1d,e deliver the same conclusion. Moreover, ABCABC-type stacking of the O layers is observed along the [010] projection.

### Imaging of Tetrahedral Li–Li Dumbbells

**Figure 2:**
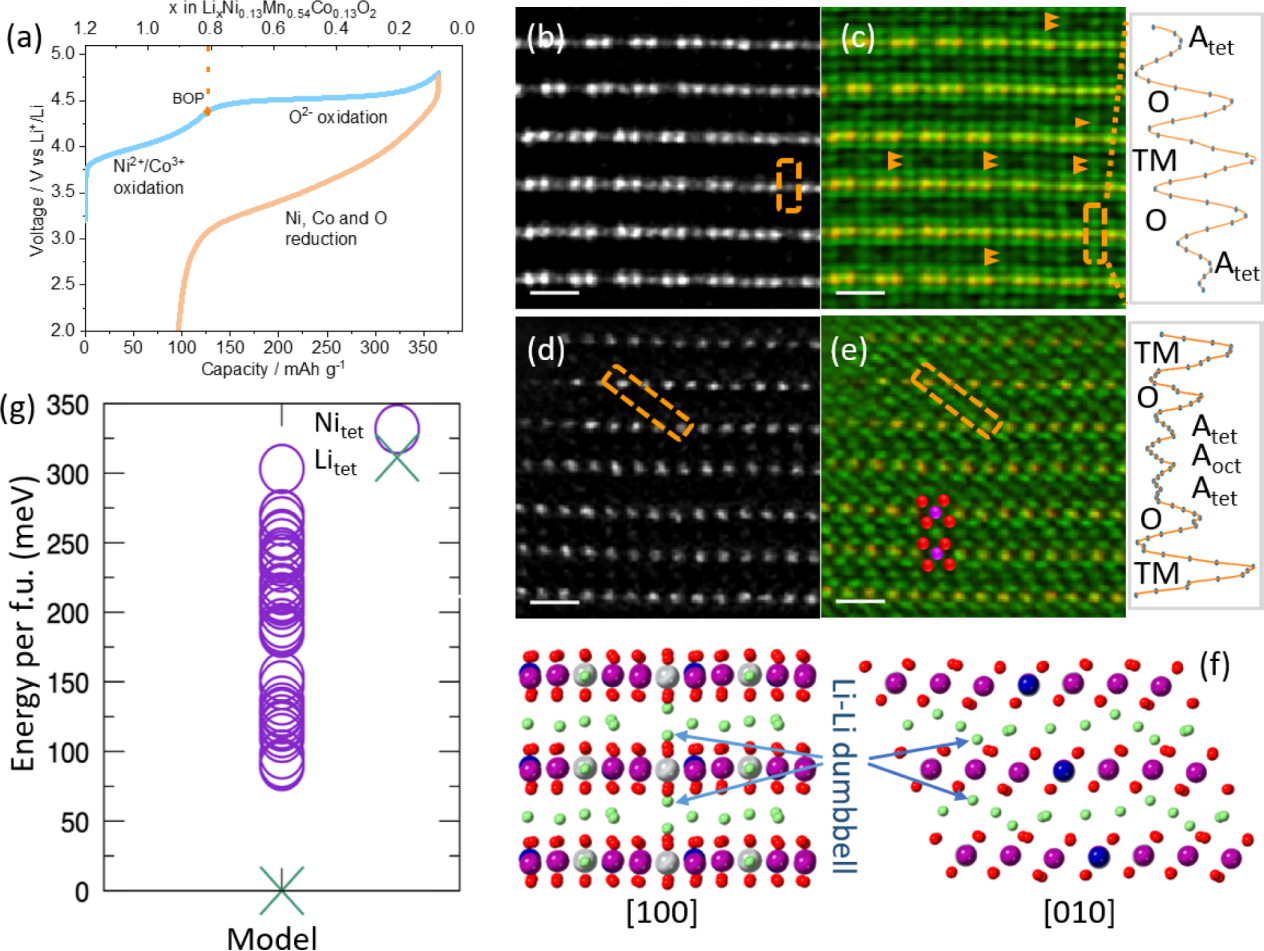
Ptychographic imaging of tetrahedral Li in Li_1.2_Ni_0.13_Mn_0.54_Co_0.13_O_2_charged to the beginning of the plateau (BOP) state. (a) First-cycle charge–discharge curve at 20 mA g^–1^ between 2 and 4.8 V. The BOP state is at a charging voltage of 4.45 V. Imaging is performed along (b,c) the mixed zone axis and (d,e) the [010] zone axis. (b,d) ADF image. (c,e) Colored ptychographic phase image. In (c), the arrows in orange indicate the tetrahedral sites showing a contrast. The highlighted region in orange starts from one alkali-layer tetrahedral site to another tetrahedral site of one adjacent alkali layer, crossing a TM layer between them. In part (e), the highlighted region in orange starts from one TM site to another TM site of the adjacent layer, crossing the alkali layer between them. In (c) and (e), the line profiles are obtained from the highlighted regions. A_tet_ indicates tetrahedral sites and A_oct_ indicates octahedral sites of alkali layers. (f) DFT model of the BOP structure projected along the [100] and [010] zone axes, respectively. The composition of the BOP model is Li_0.583_[Li_0.084_Co_0.167_Ni_0.167_Mn_0.5_]O_2_, indicating that 0.084 Li migrates from the TM layers into the alkali layers when ∼0.501 Li deintercalates from the pristine composition of Li[Li_0.1667_Co_0.167_Ni_0.167_Mn_0.5_]O_2_. (g) DFT-calculated total energies of the BOP structure with various possible configurations containing tetrahedral Ni (Ni_tet_) and Li (Li_tet_). A number of configurations of the BOP structure containing tetrahedral Ni are calculated. The energy of the configuration containing tetrahedral Li dumbbells is used as a reference energy. In (e), a local area displaying O1 stacking is superimposed with an O1 structure model. The red represents O and purple represents TMs. The scale bar of all the images is 0.5 nm.

To visualize the Li sites in Li_1.2_Ni_0.13_Mn_0.54_Co_0.13_O_2_ during the charge–discharge cycle, we charge and discharge the materials to a specific voltage in a half cell, using the Li metal as the anode, followed by cell opening and material characterization. Figure 2a plots the first charge–discharge cycle curves at 20 mA g^–1^ between 2 and 4.8 V. In the charging profile, the slope region until the BOP state is due to Ni/Co oxidation. The plateau at a higher voltage beyond the BOP state has been associated with lattice O^2–^ oxidation.^15^

For Li_1.2_Ni_0.13_Mn_0.54_Co_0.13_O_2_ in the BOP state, we characterized the structure from two distinct zone axes. Figure 2b,c display the ADF and colored ptychographic images along the mixed zone axis, and Figure 2d,e along the [010] zone axis. To minimize the surface effects arising from structure reconstruction, we selected regions of interest in the particles where there is no clear contrast from the alkali layers in the ADF images. Ptychographic reconstruction using the WDD method offers an optimized phase image with the probe focused at half of the specimen thickness.^23^ WDD optical sectioning offers 3D structural information by deconvolving the defocus aberration,^24^ a much faster data processing method compared to inverse multislice algorithms.^25^ Through optical sectioning of the ptychographic phase images at multiple defocus levels relative to the experimental focal plane, see Figure S3, we can minimize the contribution from the surface. Figure 2c displays the ptychographic phase image, and the arrows point out the alkali-layer tetrahedral sites that exhibit a contrast. The square region highlighted indicates the dumbbell contrast from the opposite tetrahedral sites relative to a TM layer. The dumbbell contrast is further demonstrated by the line profile in Figure 2c. The region showing dumbbell contrast is labeled in the simultaneous ADF image. Clearly, the ADF image does not show contrast at these sites, implying that the atoms at the tetrahedral sites are likely to be light Li atoms. In the ptychographic image of Figure 2e, the contrast from the tetrahedral sites of the alkali layers is also present (highlighted in the orange box) and further illustrated by the line profile. The octahedral site between the tetrahedral sites also shows a contrast in the ptychographic image because of the projection. Consistently, there is no visible contrast from these sites in the simultaneous ADF image of Figure 2d.

To understand the observed tetrahedral contrast, we performed DFT calculations to predict the structure of the BOP Li_1.2_Ni_0.13_Mn_0.54_Co_0.13_O_2_. A composition of Li[Li_0.1667_Co_0.167_Ni_0.167_Mn_0.5_]O_2_ is used for the computation as an initial structure, which is a closely related compound to the synthesized Li_1.2_Ni_0.13_Mn_0.54_Co_0.13_O_2_, see the model in Figure 1a. The species in parentheses are located in the TM layers. The initial BOP model is obtained by setting the Co and Ni ions at the 4+ state and removing ∼Li_0.501_ from the alkali layers since removing Li^+^ ions from the alkali layers is preferential compared with that from the TM layers, consistent with the previous reports.^19^ By relaxation of this initial model, the most stable BOP structure at the lowest energy is found and is shown in Figure 2f. DFT calculations, see Supplementary Video 1, indicate that in forming this energy-favorable BOP model, a TM-layer Li^+^ cation spontaneously migrates out of the TM layer into one tetrahedral site of the alkali layers, immediately followed by the migration of an octahedral Li^+^ cation from the alkali layers to the opposite tetrahedral site across a TM-layer vacancy, forming a Li–Li dumbbell configuration. The tetrahedral Li in the alkali layers is trapped at a local energy minimum and does not migrate to the neighboring octahedral site immediately, see Supplementary Video 2. We also compute that it is more favorable for about half of the TM-layer Li to migrate into the tetrahedral sites, giving a composition of Li_0.583_[Li_0.084_Co_0.167_Ni_0.167_Mn_0.5_]O_2_. Moreover, we observe that the Li^+^ ions in the alkali layers are slightly off the octahedral center, see the scheme in Figure 2f and the polyhedral model in Figure S4.

Since the Ni^2+^ ions in Li_1.2_Ni_0.13_Mn_0.54_Co_0.13_O_2_ can migrate from one octahedral site of the TM layers to another octahedral site of the alkali layers through an empty tetrahedral site with relatively low energy barriers,^19^ the tetrahedral sites might be occupied by Ni. To examine this further, we computed the formation energy of a number of models with Ni at the tetrahedral sites instead of Li, to evaluate the structural stability. Figure 2g plots the formation energy of the models with various configurations of tetrahedral Ni in BOP Li_1.2_Ni_0.13_Mn_0.54_Co_0.13_O_2_, relative to the formation energy of tetrahedral Li that is used as a reference energy. The tetrahedral Ni models are always at energies higher than the tetrahedral Li model depicted in Figure 2f, indicating that the latter is the most stable BOP structure.

Through the ptychographic imaging on BOP Li_1.2_Ni_0.13_Mn_0.54_Co_0.13_O_2_, we demonstrate that there are light atoms occupying the alkali-layer tetrahedral sites, and the atoms are likely to be Li. The observed structure is found to be highly consistent with the DFT models of the BOP structure depicted in Figure 2f. Therefore, the atoms at the tetrahedral sites are Li, rather than TMs. Identifying Li at these tetrahedral sites at an atomic resolution is only possible using the ptychography data. The commonly used ADF imaging^26^ and spectroscopy^27^ techniques, which can identify the atom species struggle to determine the species of the tetrahedral atoms. The reasons are that the atoms at the tetrahedral site are invisible in ADF, and atomic-scale spectroscopy requires high electron doses, which can cause serious beam damage to the sample and rarely succeeds for battery materials. In the next paragraph, we will demonstrate how to mathematically process the quantitative ptychographic phase images and use the resulting quantities to identify the atoms and, in particular, to identify them as Li located at the tetrahedral sites of BOP Li_1.2_Ni_0.13_Mn_0.54_Co_0.13_O_2_.

**Figure 3:**
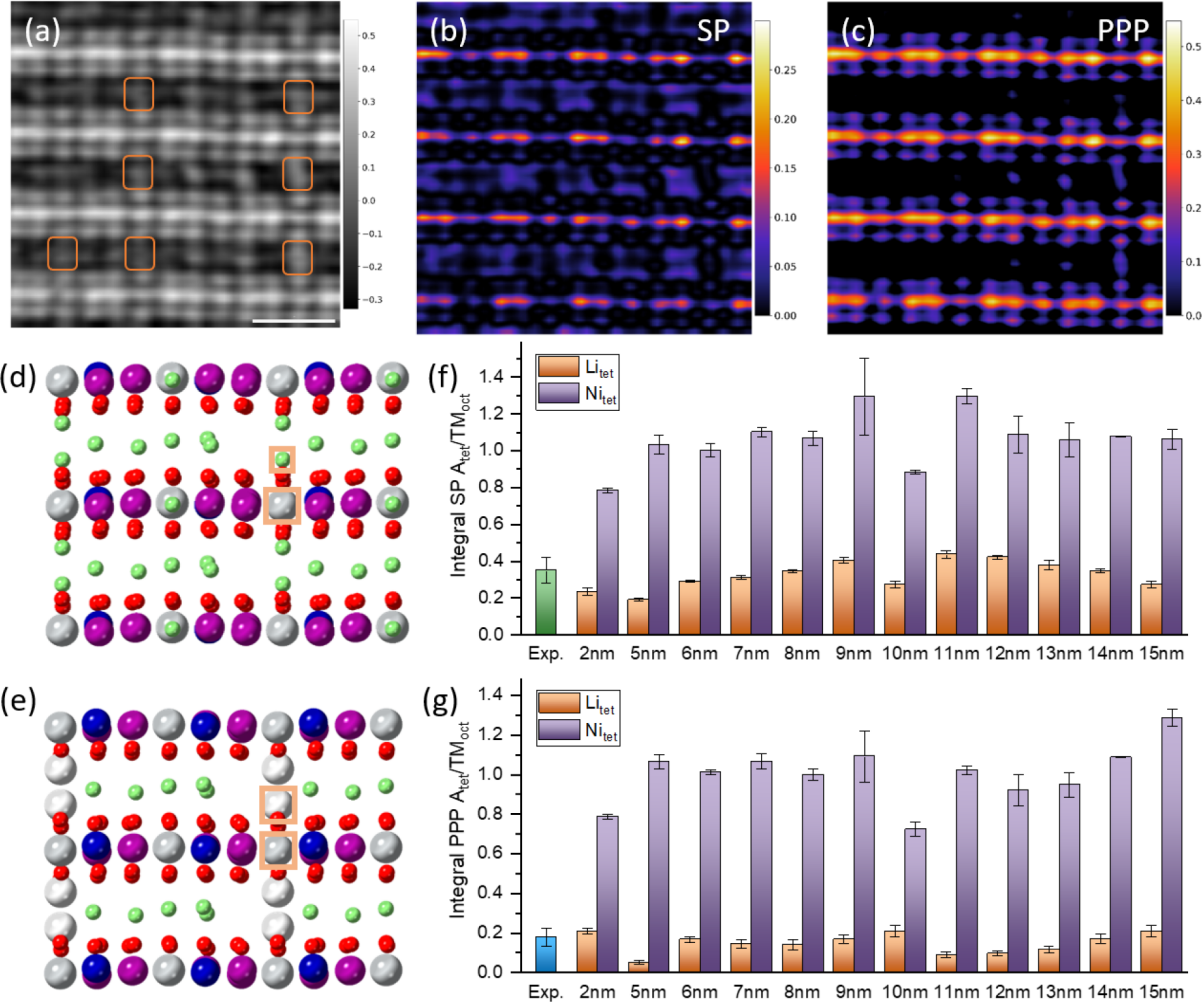
Quantification of ptychographic phase contrast of BOP Li_1.2_Ni_0.13_Mn_0.54_Co_0.13_O_2_. (a) Ptychographic phase image marked with alkali layers showing a dumbbell contrast. The scale bar is 0.5 nm. The phase is mathematically processed by squaring the phase or by extracting the partial positive phase. (b) Squared phase (SP) image. (c) Partial positive phase (PPP) image. The SP and PPP values around each atom are integrated using Voronoi cells. The ratios of the integral SP and PPP values from the alkali-layer tetrahedral sites (A_tet_) as a fraction of those from the TM-layer octahedral sites (TM_oct_) are calculated. The tetrahedral and octahedral sites are indicated by the boxes over the two DFT models: (d) tetrahedral Li model with Li ions at the tetrahedral sites and (e) tetrahedral Ni model with Ni ions at the tetrahedral sites. In the models, gray is Ni, purple is Mn, blue is Co, red is O, and green is Li. The ratios from the experimental image are compared to those from the simulated images of the two DFT models of a range of thicknesses. Plots of the ratios were obtained using the (f) integral SP values and (g) integral PPP values.

Figure 3a shows a ptychographic image of BOP Li_1.2_Ni_0.13_Mn_0.54_Co_0.13_O_2_ along the mixed zone axis. The alkali-layer tetrahedral sites showing contrast are highlighted in orange. Although the ptychographic phase is quantitative, the positive and negative values in the ptychographic image point spread function^28^ exclude direct phase integration over the atoms to correlate with the scattering cross section of specific atoms.

Here, we developed three methods to process the ptychographic phase to overcome the inherent negative regions of ptychographic images to allow direct integration: (i) to square the phase to form squared phase (SP) images, see Figure 3b; (ii) to extract the positive phase and change all the negative phase to 0, resulting in partial positive phase (PPP) images, see Figure 3c; and (iii) to lift the phase minimum to 0, resulting in positive phase (PP) images, see Figure S5. To integrate the SP, PPP, and PP values over each atom, we first determine the atom positions over the ptychographic images using the center of mass, followed by peak fitting using 2D Gaussian functions. Then, the SP, PPP, and PP values are integrated over the refined atom positions using Voronoi cells, resulting in integral values of each atom column. Figure S6 shows the integral SP and PPP values, and Figure S5 shows the integral PP values over each atom column shown in the ptychographic image of BOP Li_1.2_Ni_0.13_Mn_0.54_Co_0.13_O_2_. The protocols of ptychographic image processing and quantification are schematically shown in Figure S7.

To identify the atoms at the tetrahedral sites of BOP Li_1.2_Ni_0.13_Mn_0.54_Co_0.13_O_2_, we calculated the ratios of the integrated values of the alkali-layer tetrahedral sites (A_tet_) relative to the nearest TM-layer octahedral sites (TM_oct_) and compared them with the results obtained from the simulated images of the DFT-calculated models. Figure 3d shows the tetrahedral Li model with Li–Li dumbbells at the opposite tetrahedral sites of the alkali layers, and Figure 3e displays the tetrahedral Ni model with Ni–Ni dumbbells. In the images, the marked sites represent where the ratios are calculated. Figure S8 shows the simulated ptychographic images of the tetrahedral Li model of different thicknesses, and Figure S9 shows the images of the tetrahedral Ni model. All the simulated images are also processed using the above three routes.

Figure 3f plots the integral SP ratios obtained from the experimental ptychographic image and simulated ptychographic image of the tetrahedral Li and tetrahedral Ni model. Figure 3g displays the results of the integral PPP ratios, and Figure S4 shows the results of the integral PP ratios. We found that the integral SP and PPP ratios from the experimental ptychographic image correspond with those from the simulated images of the tetrahedral Li model but cannot match those from the tetrahedral Ni model. The integral PP ratios from the experimental image coincide with most of those of the tetrahedral Li model but not the tetrahedral Ni model. We conclude that the mathematical processing of the ptychographic phase using SP and PPP methods is capable of distinguishing Li and Ni. The observed contrast from the tetrahedral sites of the BOP Li_1.2_Ni_0.13_Mn_0.54_Co_0.13_O_2_ is from Li, rather than Ni, nor Mn and Co, which have a similar *Z* number to Ni.

Many other techniques are also performed to demonstrate that the tetrahedral sites are occupied by Li rather than Ni. For example, we carried out multiframe low-dose energy-dispersive X-ray spectroscopy (EDX) on BOP Li_1.2_Ni_0.13_Mn_0.54_Co_0.13_O_2_ at the atomic scale. The results indicate the absence of metals at the tetrahedral sites; see Figure S10, supporting the conclusion that Li ions are at the tetrahedral sites. Furthermore, we simulated the neutron pair distribution function (PDF) with a Li–Li partial function (Figure S11a) and a full function (Figure S11b) using the DFT models of Li_1.2_Ni_0.13_Mn_0.54_Co_0.13_O_2_. A new peak at 3.2 Å is observed for the BOP model, arising from the correlation distance between the alkali-layer tetrahedral Li and the nearest TM-layer octahedral TMs. This peak can be observed in the reported neutron PDF of Li_1.2_Ni_0.13_Mn_0.54_Co_0.13_O_2_ charged to 4.4 V^29^ but has not been discussed. Moreover, the TMs in BOP Li_1.2_Ni_0.13_Mn_0.54_Co_0.13_O_2_ are highly oxidized and reported to be unstable at the tetrahedral sites, according to previous calculations.^30^ Herein, we have deployed the quantitative ptychographic phase through specific mathematical processing, together with DFT calculations, EDX, and neutron PDF, demonstrating that there are Li^+^ ions of the TM layers migrating into the tetrahedral sites of Li_1.2_Ni_0.13_Mn_0.54_Co_0.13_O_2_ in the charge to the BOP state.

### Stability of Tetrahedral Li

Despite the preserved O3-stacking O layers in BOP Li_1.2_Ni_0.13_Mn_0.54_Co_0.13_O_2_, a region of O1 phase with ABAB-type O stacking is observed, see Figure 2e. The O3 to O1 phase change is a typical structural evolution in layered metal oxides, resulting from the layer gliding during Li deintercalation.^31^ Multiple images of BOP Li_1.2_Ni_0.13_Mn_0.54_Co_0.13_O_2_ in Figure S12 with a field of view of ∼52 nm^2^ indicate that the amount of the O1 phase is relatively low compared with the O3 phase. What is striking in these images is that the contrast from tetrahedral Li is observed in the O3 phase but is absent in the O1 phase; see Figure 2e. We performed DFT calculations to explore the underlying reasons.

**Figure 4:**
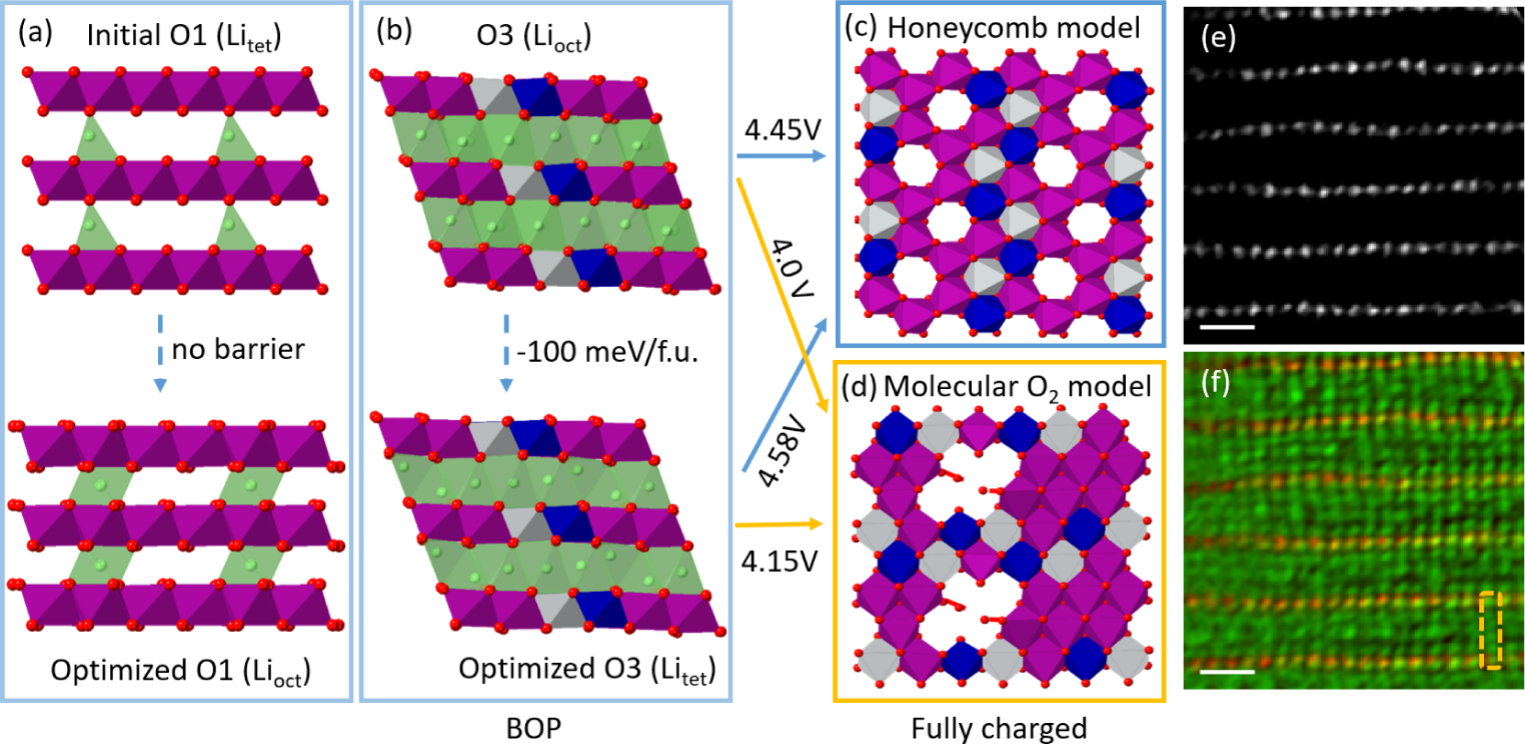
Stability of tetrahedral Li in Li_1.2_Ni_0.13_Mn_0.54_Co_0.13_O_2_. (a) DFT models of an initial O1 structure with Li ions placed at the tetrahedral sites, which move into the octahedral sites after relaxation. The initial O1 model only considers Li at the tetrahedral sites. (b) DFT models of the O3 structure in the BOP state with Li ions in alkali layers located at the octahedral sites, and with partial Li at the tetrahedral sites. The latter displays lower energy by 100 meV/formula unit (f.u.). (c) Honeycomb model and (d) molecular O_2_ models of the fully charged state are considered by referring to our previous work.^21^ Removing all the Li ions from the BOP state to the fully charged state requires high-voltage charging. (e) ADF image and (f) colored ptychographic phase image of Li_1.2_Ni_0.13_Mn_0.54_Co_0.13_O_2_ at a charging voltage of 4.8 V.

Figure 4a shows the initial DFT model of an O1 layered structure with Li ions placed at the alkali-layer tetrahedral sites. The Li ions are found to migrate to the octahedral sites without an energy barrier after model relaxation. Thus, the O1 phase is not thermodynamically able to accommodate Li at the tetrahedral sites. In contrast, we demonstrated in Figure 2 that the O3 phase containing tetrahedral Li is a stable structure at the BOP state. In the optimized DFT model of the O1 phase, the O-layer spacing of the alkali layers is 2 Å, while the spacing of the O3 phase at the BOP state is 2.86 Å; see Figure S13. This result is consistent with the experimental data. We measured the projected axial O–O distance in BOP Li_1.2_Ni_0.13_Mn_0.54_Co_0.13_O_2_, and the O3 phase shows longer values than the O1 phase, see Figure S14. The more spacious layer spacing of the O3 phase is likely to be a reason why Li can be stable at the tetrahedral sites. Indeed, Li conduction across the alkali layers undergoes the *o*–*t*–*o* channels, and a large layer spacing can significantly lower the activation barrier for Li migration via the tetrahedral sites,^2,3,5^ supporting our conclusions.

We then investigate how the tetrahedral Li in the O3 BOP Li_1.2_Ni_0.13_Mn_0.54_Co_0.13_O_2_ impacts the electrochemical performance. In Figure 4b, the O3 structure containing tetrahedral Li is observed to have a much lower formation energy by 100 meV/formula unit than that without tetrahedral Li, therefore requiring a higher voltage to deintercalate the Li ions. To quantitatively understand the voltage, we computed the average voltage in deintercalating the Li ions from the BOP Li_1.2_Ni_0.13_Mn_0.54_Co_0.13_O_2_ to the fully charged state using the Nernst equation. We considered two fully charged models which have been discussed in our previous work.^21^ The first one is the honeycomb model, see Figure 4c, with electron holes pinned on the oxide anions, and the other one is the molecular O_2_ model, see Figure 4d, with the formation of the O_2_ molecules trapped in the TM-layer vacancies. Our calculations show that the O3 phase with tetrahedral Li requires a 4.15 V voltage plateau to reach the molecular O_2_ model and a 4.58 V one is needed for the honeycomb model. Thus, the former model with molecular O_2_/TM vacancies is more plausible to occur beyond the BOP state, that is, in the O oxidation process. Indeed, resonant inelastic X-ray scattering^15^ and neutron PDF^32^ studies have demonstrated such a molecular O_2_ model structure in O oxidation. In comparison with the calculated voltages of 4.15 and 4.58 V, the electrochemically tested equilibrium voltage of O oxidation of Li_1.2_Ni_0.13_Mn_0.54_Co_0.13_O_2_ is found to be below 4.5 V^33,34^ implying that the O oxidation is a nonequilibrium process and leads to a mixed structure of both fully charged models.

Deintercalating the tetrahedral Li of BOP Li_1.2_Ni_0.13_Mn_0.54_Co_0.13_O_2_ is anticipated by the performed charging cutoff of 4.8 V. Figure 4e,f show the ADF and colored ptychographic image of the fully charged Li_1.2_Ni_0.13_Mn_0.54_Co_0.13_O_2_ at 4.8 V. The tetrahedral Li^+^ ions observed in BOP Li_1.2_Ni_0.13_Mn_0.54_Co_0.13_O_2_ are much more rarely observed, suggesting deintercalation of the tetrahedral Li, which occurs below the reported voltage of 5 V.^19^Figure 4f also shows residual tetrahedral Li, likely in a very small amount within this limited field of view. The first-cycle charge profile of Li_1.2_Ni_0.13_Mn_0.54_Co_0.13_O_2_ has indicated that there is ∼0.1 Li per formula unit still left in the structure by 4.8 V. The tetrahedral Li could be part of this residual Li in fully charged Li_1.2_Ni_0.13_Mn_0.54_Co_0.13_O_2_. Additionally, the alkali layers in the fully charged state cannot be vacuumed but contain a complex mixture of vacancies, residual Li^+^ ions, and migrating cations, leading to a variable contrast in the phase images.

### Reintercalation of Tetrahedral Li

**Figure 5:**
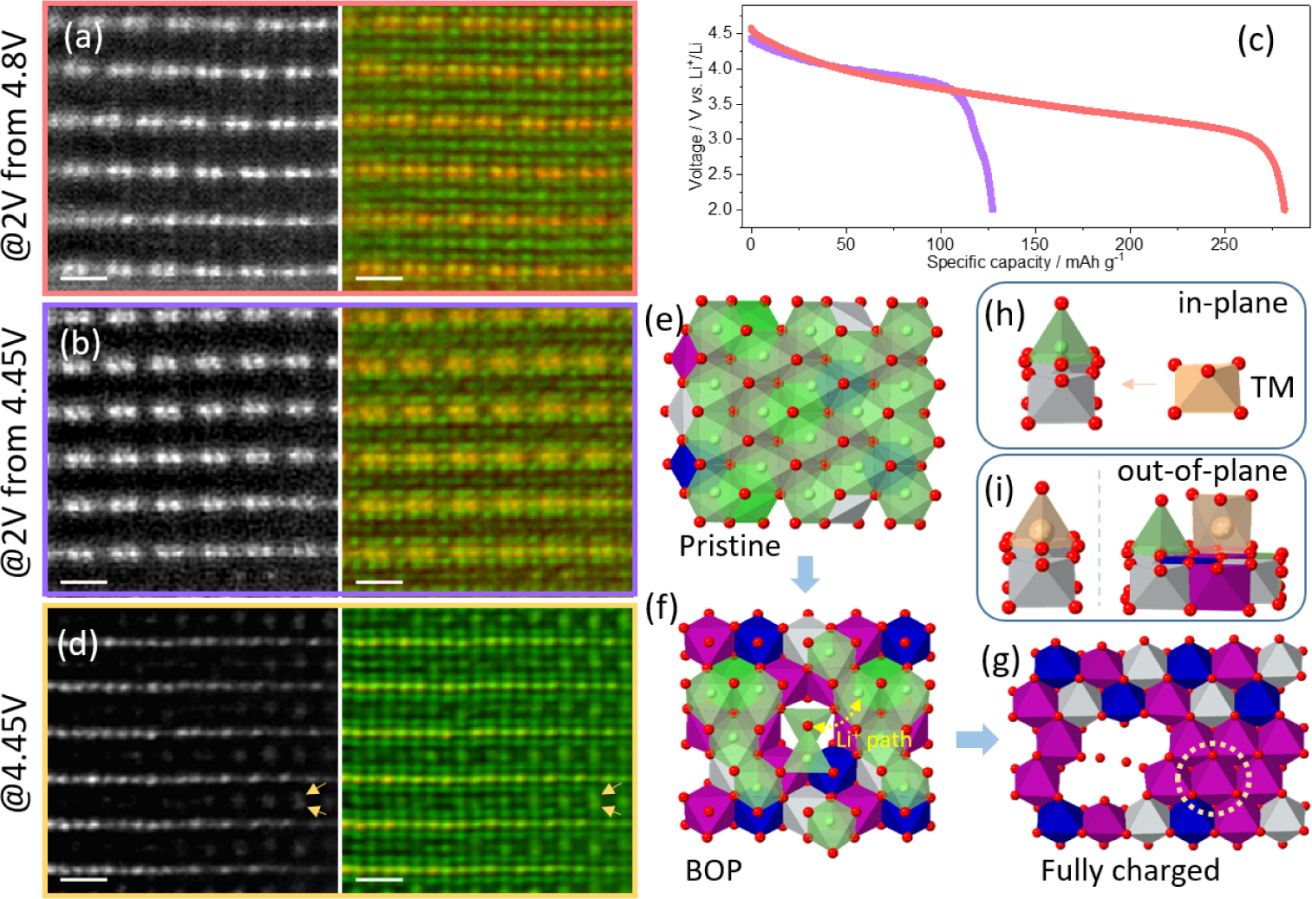
Loss of tetrahedral Li in Li_1.2_Ni_0.13_Mn_0.54_Co_0.13_O_2_. The images in a,b,d show the ADF image (left) and colored ptychographic phase image (right). The projection is along the mixing zone axis. The scale bar is 0.5 nm. (a) At the discharged voltage of 2 V after charging to 4.8 V. (b) At the discharged voltage of 2 V after charging to 4.45 V. (c) First-cycle discharge curve after charging to 4.8 and 4.45 V at a current density of 20 mA g^–1^. (d) Images showing clear TM migration at 4.45 V. The ADF images show clear in-plane and out-of-plane TM migration. The yellow arrows mark the TMs at the alkali-layer tetrahedral sites, forming TM–TM dumbbell contrast. (e–g) Scheme of Li migration in charging to the BOP and fully charged state, viewed perpendicular to the planes. In the models, purple represents Mn, blue Co, gray Ni, and red O. Green represents Li ions in the alkali layers, while light green depicts Li ions of TM layers. Scheme of the (h) in-plane TM migration and (i) out-plane TM migration to the alkali-layer tetrahedral and octahedral sites, viewing slightly off parallel to the planes. The bottom layer is the TM layer, and above is the nearest alkali layer.

Whether the tetrahedral Li can be reintercalated into the TM layers of Li_1.2_Ni_0.13_Mn_0.54_Co_0.13_O_2_ is highly associated with the reversibility of the capacity and voltage. To investigate the reintercalation of the tetrahedral Li, we characterized the fully discharged Li_1.2_Ni_0.13_Mn_0.54_Co_0.13_O_2_ at 2 V from different charging cutoffs of 4.8 and 4.45 V, respectively. Figure 5a shows the ADF and colored ptychographic image of the fully discharged Li_1.2_Ni_0.13_Mn_0.54_Co_0.13_O_2_ from 4.8 V and Figure 5b from 4.45 V. The discharge profiles are plotted in Figure 5c. The ADF images of both discharged samples present a distinguishable TM–TM dumbbell contrast, suggesting the honeycomb ordering of the TM layers. The ptychographic images, also see Figure S14 in different regions, show absent contrast from the tetrahedral sites, indicating the disappearance of the tetrahedral Li after discharging. NMR studies have reported that the TM layers of Li_1.2_Ni_0.13_Mn_0.54_Co_0.13_O_2_ can be reintercalated by Li^+^ ions after discharging.^15^ We believe that the tetrahedral Li of the BOP and fully charged Li_1.2_Ni_0.13_Mn_0.54_Co_0.13_O_2_ have migrated into the TM layers during discharge, regardless of the charging cutoffs.

### Stability of Tetrahedral Li After TM Migration

In-plane and out-of-plane TM migration are typical structural changes in Li_1.2_Ni_0.13_Mn_0.54_Co_0.13_O_2_ during cycling.^21^ To discuss how TM migration affects the stability of tetrahedral Li, we showed a close-to-surface region of BOP Li_1.2_Ni_0.13_Mn_0.54_Co_0.13_O_2_ in Figure 5d, where both in-plane and out-of-plane TM migration have occurred. The in-plane TM migration disrupts the honeycomb cation ordering and leads to the TM–TM dumbbell contrast disappearing in the ADF image. The out-of-plane TM migration causes the TMs to migrate into the alkali layers and occupy the vacancies left by the deintercalated Li^+^ ions. The arrows in yellow show tetrahedral sites displaying a contrast in the ADF and ptychographic images. The high contrast in ADF indicates that these tetrahedral sites are occupied by TMs, which has been observed in a Mn_3_O_4_-like spinel.^35^ In this close-to-surface region displayed in Figure 5d, the simultaneous ADF and ptychographic images show no evidence of tetrahedral Li, unlike that indicated by Figure 2.

We propose explanations for the disappearance of tetrahedral Li in BOP Li_1.2_Ni_0.13_Mn_0.54_Co_0.13_O_2_ when TM migration occurs. Figure 5e–g schematically depicts the Li_1.2_Ni_0.13_Mn_0.54_Co_0.13_O_2_ structure from the pristine to the BOP state and to the fully charged state within an O3 phase. Figure 5h,i schematically show the in-plane and out-of-plane TM migration into the octahedra face-sharing with a tetrahedron in alkali layers.

As discussed previously^19^ the TM-layer Li^+^ ions migrate to the alkali layers and form tetrahedral Li–Li dumbbells structurally in BOP Li_1.2_Ni_0.13_Mn_0.54_Co_0.13_O_2_, as can be seen in the experimental observations in Figure 2. We noticed that in the alkali layers of Li_1.2_Ni_0.13_Mn_0.54_Co_0.13_O_2_, a tetrahedron shares four faces with four octahedra; three of them are in the alkali layers containing Li, and one of them is in the TM layers. Our calculations shown in Figure 2, which are consistent with previous results,^19,36^ suggest that the tetrahedral Li can be a stable site in BOP Li_1.2_Ni_0.13_Mn_0.54_Co_0.13_O_2_ if the face-sharing octahedral sites are empty, reducing the electrostatic interaction and steric effects.^3,37,38^

Because the O^2–^ oxidation in Li_1.2_Ni_0.13_Mn_0.54_Co_0.13_O_2_ beyond the BOP state can lead to vacancy clustering in the TM layers,^15^ the atoms at the tetrahedral sites face less repulsion when they are close to the vacancy clusters than when they are close to TMs, see the schematic representation in Figure 5g. Previous theoretical calculations suggest that Li situated in the tetrahedral site can experience repulsion from its nearby TMs, and the repulsive force intensifies with the increase in the occupancy of the face-sharing octahedra and the valence states of the TMs.^3^ The repulsion may explain the lack of observed tetrahedral Li due to the site now becoming unstable for Li occupation. The tetrahedral sites close to the vacancy clusters may be the main accommodation sites for the residual tetrahedral Li in fully charged Li_1.2_Ni_0.13_Mn_0.54_Co_0.13_O_2_, as observed in Figure 4f.

The TM migration in Li_1.2_Ni_0.13_Mn_0.54_Co_0.13_O_2_ is not observed to be fully reversible after discharging.^15,21^ When these migrated TMs occupy the octahedral sites face-sharing with the alkali-layer tetrahedral sites, the repulsion between the octahedral and tetrahedral sites can persist and destabilize Li at the tetrahedral sites. If the repulsion is considerable, then the Li diffusion could be blocked, preventing the vacant Li sites from being occupied. The Li vacancies in the TM layers that cannot be filled on discharging may enhance the in-plane TM migration, facilitating further O_2_ formation and contributing to voltage fade.^15,18^

## Conclusions

In this work, by employing a combination of electron ptychography, ADF imaging, and DFT calculations, we have demonstrated the presence of tetrahedral Li in Li_1.2_Ni_0.13_Mn_0.54_Co_0.13_O_2_ after the TM-redox region of the first cycle at the BOP state. We were able to rule out the possible occupancy of tetrahedral sites by TMs through quantitative analysis of the ptychographic phase images from atomic resolutions, reinforcing the conclusion that these tetrahedral sites are occupied by Li. The tetrahedral Li forms Li–Li dumbbells with Li occupying the opposite tetrahedral sites face-sharing with an octahedral site of the TM layer containing a Li vacancy. During further delithiation beyond the BOP state, most of the tetrahedral Li is removed, leaving only a small amount of residual tetrahedral Li at the fully charged state. The delithiation process is also accompanied by in- and out-of-plane TM migration and a phase change from O3 to O1, which we show cause some of the tetrahedral Li sites to become unstable, explaining the reducting amount of tetrahedral Li observed. On discharge, irreversible out-of-plane TM migration into the Li layer disfavors the occupancy of some alkali-layer tetrahedral sites by Li and the repopulation of some vacant TM sites, potentially contributing to capacity loss and sluggish Li reinsertion. To maintain the maximum number of accessible Li sites and minimize the blocking of tetrahedral Li diffusion pathways, strategies to mitigate the irreversible TM migration and O1 phase change should be employed.

## Experimental Procedures

### Material Synthesis

We used the sol–gel method for the synthesis of Li_1.2_Mn_0.54_Co_0.13_Ni_0.13_O_2_ as reported previously.^21^ To be specific, stoichiometric amounts of LiCH_3_COO·2H_2_O (99.0%, Aldrich), Co(CH_3_COO)_2_·4H_2_O (99.0%, Aldrich), Ni(CH_3_COO)_2_·4H_2_O (99.0%, Aldrich), and Mn(CH_3_COO)_2_·4H_2_O (99.0%, Fluka) were dissolved in 50 mL of distilled water. A separate mixture containing 0.1 mol of resorcinol (99.0%, Fluka), 0.15 mol of formaldehyde (Fluka 36.5% in water, methanol-stabilized), and 0.25 mmol of Li_2_CO_3_ (99.0%, Fluka) was also made. Both solutions were stirred until all the reagents were dissolved, before being added together and stirred for 30 min. A 5 mol % excess of Li was used. The mixture was then dried at 90 °C for 8 h, followed by burning the organic matter at 480 °C for 15 h. The fine powder was removed from the furnace, ground, and calcined at 900 °C for 15 h to obtain the final material.

### Electrochemical Characterization

The cathode was prepared by mixing 80 wt % active material of the as-prepared Li_1.2_Ni_0.13_Mn_0.54_Co_0.13_O_2_, 10 wt % Super P conductive carbon, and 10 wt % polytetrafluoroethylene binder in a mortar and rolling it into a film. CR2032 coin cells were assembled using the prepared cathode, glass fiber separators, 1 M LiPF_6_ in EC/DMC (v/v = 1:1) as the electrolyte, and lithium foil as the anode. The current density used was 20 mA g^–1^, with a voltage window of 2 to 4.8 V being used for cycling.

### Specimen Preparation and TEM Transfer

The as-assembled coin cells were charged and discharged to a specific voltage and opened in an Ar-filled glovebox. The cathode material was collected from the cells and washed with DMC solvent in the glovebox, followed by sonication. The obtained solution dispersion was dropped on the TEM grids, followed by drying in the glovebox. The TEM grids were then mounted on the JEOL anaerobic transfer TEM holder in the glovebox, followed by transfer to the microscope for imaging.

### TEM Data Collection

Electron microscopy studies were carried out on an aberration-corrected JEOL ARM200F electron microscope operated at 200 kV. In ADF-STEM imaging, the convergence semiangle is 22.4 mrad, and the inner to outer collection angle is 72.8–271 mrad. In this setup, the depth of focus (Δ*Z*) along the optical axis is about 8.5 nm (Δ*Z* = 1.7λ/α^2^ where λ is the electron wavelength and α is the convergent semiangle of the probe). Multiframe low-dose EDX spectroscopy was carried out on the ePSIC E01 instrument (ARM200F mounted with large solid angle dual EDX detectors) at Diamond Light Source using the on-site control software. The data were aligned and reconstructed using the in-house Python codes. In recording the 4D STEM data set, a focused probe is used, and the 2D convergent-beam electron diffraction (CBED) pattern at each probe position was recorded using a JEOL 4Dcanvas pixelated detector mounted on the ARM200F. The camera has 264 × 264 physical pixels, and through pixel binning by 4-fold (64 × 264 pixels), the recording speed can reach ∼7.5 k frames per second. We use this fastest speed mode to record the 4D data sets used in this work. The real-space 2D raster scanning contains 256 × 256 pixels, resulting in the collection of 256 × 256 frames of 2D diffraction patterns in ∼8.7 s. The emission current of the electron gun is down to 5 μA or below to avoid saturation of the pixelated detector while lowering the beam damage. The probe current is below 11.4 pA. The dose of each data set depends on the real-time probe current and pixel size. The aberration-corrected ADF images were recorded simultaneously using the JEOL ADF detector with a collection of 2D diffraction patterns. The simultaneous recording of ADF and 4D STEM data sets was limited by the speed of the pixelated detector and can cause scan distortion and sample drift during data acquisition. In imaging the particles, we treat the regions undergoing cation mixing and cubic structure reconstruction during the charge–discharge cycling as the surface, and the regions beyond the reformed surface as bulk.

### Electron Ptychography Reconstruction

Ptychographic phase reconstruction from the 4D STEM data set of the sample is a postprocessing of the entire CBEDs. The Wigner distribution deconvolution (WDD) algorithm is one of the commonly used methods to retrieve phase information. In the reconstruction, the residual aberrations in the data sets can also be corrected by numerical processing. To do this, an optimized probe function is determined by measuring the aberrations using the singular value decomposition (SVD) algorithm. Aberration-free phase images are then reconstructed by correcting the aberrations.

### Density Functional Theory (DFT)

Spin-polarized calculations were conducted using the Quantum Espresso package.^39^ The Perdew, Burke, and Ernzerhof (PBE) exchange-correlation function was employed.^40^ The core–valence interaction was described via norm-conserving pseudopotentials.^41^ The wave functions were represented via plane-wave basis sets with an energy cutoff of 120 Ry. Hubbard corrections (DFT+U) were included to correctly describe the energetics of the 3d orbitals of the transition metals (TM), with *U* = 4, 6, and 5 eV for Mn, Ni, and Co, respectively. A 2 × 2 × 4 Monkhorst–Pack *k*-point grid was used. Crystal structures were relaxed until forces on the atoms were less than 0.02 eV/Å and total stresses on the cell were less than 0.05 kBar. On top of the DFT+U simulations, we performed hybrid calculations employing the Heyd–Scuseria–Ernzerhof (HSE) functional,^39^ with an exact exchange mixing parameter of 0.25. The input structures were obtained from the DFT+U optimizations, and the atomic positions were allowed to relax further at the HSE level, keeping the lattice parameters fixed. A correction of 1.06 eV/O_2_ was introduced to calculate formation energies, to remediate the overestimation of the binding energy of O_2_ originating from the use of the HSE functional.^42,43^ A supercell of the closely related compound Li[Li_0.1667_Co_0.167_Ni_0.167_Mn_0.5_]O_2_, which contains 96 atoms (28 Li atoms; 12 Mn atoms; 4 Ni atoms; 4 Co atoms; 48 O atoms), was optimized and used as an initial structure to investigate charged and discharged states. To study the in-plane TM disorder in the charged state, structural models with different TM distributions in the TM layers were prepared using combinatorics. The same strategy was used to investigate Li ordering. The simple random sampling (SRS) method was used to choose a representative subset of configurations for relaxation. To model the relative stability of tetrahedral Li in O1- and O3-type layered structures, we considered the layered material Li_2_MnO_3_ as a model cathode. The BOP model of Li[Li_0.1667_Co_0.167_Ni_0.167_Mn_0.50_]O_2_ is obtained by setting the Co and Ni at the 4+ state and removing the corresponding amount of Li from the alkali layers. There will be Li migrating from the TM layers into the alkali layers in this process, resulting in a unit formula of Li_0.583_[Li_0.084_Co_0.167_Ni_0.167_Mn_0.5_]O_2_. The BOP models with and without tetrahedral Li have the same unit formula. The fully charged model is obtained by removing all of the Li out of the structure. Voltage profiles from the BOP state to the fully charged state were computed using the Nernst equation, as detailed in ref (11).

## Supporting Information Available

ADF images (Figure S1); ptychographic phase images (Figure S2); WDD optical sectioning (Figure S3); schematic representation of tetrahedral Li–Li dumbbells (Figure S4); mathematical processing of the ptychographic phase (Figure S5); integration of the values in SP and PPP images (Figure S6); protocols for quantitative use of the ptychographic phase (Figure S7); simulated ptychographic images of the tetrahedral Li model (Figure S8); simulated ptychographic images of the tetrahedral Ni model (Figure S9); EDX spectroscopy (Figure S10); simulated neutron PDF of LRNMC (Figure S11); colored ptychographic phase image (Figure S12); DFT calculation results (Figure S13); atom distance measurements (Figure S14) (PDF)
Li migration from metal-layer octahedral sites to alkali-layer tetrahedral sites (MP4)
Trapping of tetrahedral Li in the alkali layers at a local energy minimum (MP4)

## References

[ref1] Ji H.; Wu J.; Cai Z.; Liu J.; Kwon D.-H.; Kim H.; Urban A.; Papp J. K.; Foley E.; Tian Y.; Balasubramanian M.; Kim H.; Clément R. J.; McCloskey B. D.; Yang W.; Ceder G. Ultrahigh power and energy density in partially ordered lithium-ion cathode materials. Nat. Energy 2020, 5, 213–221. 10.1038/s41560-020-0573-1.

[ref2] Kang K.; Ceder G. Factors that affect Li mobility in layered lithium transition metal oxides. Phys. Rev. B 2006, 74, 094105 10.1103/PhysRevB.74.094105.

[ref3] Kang K.; Meng Y. S.; Breger J.; Grey C. P.; Ceder G. Electrodes with high power and high capacity for rechargeable lithium batteries. Science 2006, 311, 977–980. 10.1126/science.1122152. 16484487

[ref4] Wei Y.; Zheng J.; Cui S.; Song X.; Su Y.; Deng W.; Wu Z.; Wang X.; Wang W.; Rao M.; Lin Y.; Wang C.; Amine K.; Pan F. Kinetics Tuning of Li-Ion Diffusion in Layered Li(Ni_x_Mn_y_Co_z_)O_2_. J. Am. Chem. Soc. 2015, 137, 8364–8367. 10.1021/jacs.5b04040. 26098282

[ref5] Catti M. Ab initio study of Li^+^ diffusion paths in the monoclinic Li_0.5_CoO_2_. Phys. Rev. B 2000, 61, 1795–1803. 10.1103/PhysRevB.61.1795.

[ref6] Goodenough J. B.; Kim Y. Challenges for Rechargeable Li Batteries†. Chem. Mater. 2010, 22, 587–603. 10.1021/cm901452z.

[ref7] Assat G.; Tarascon J.-M. Fundamental understanding and practical challenges of anionic redox activity in Li-ion batteries. Nat. Energy 2018, 3, 373–386. 10.1038/s41560-018-0097-0.

[ref8] Seo D.-H.; Lee J.; Urban A.; Malik R.; Kang S.; Ceder G. The structural and chemical origin of the oxygen redox activity in layered and cation-disordered Li-excess cathode materials. Nat. Chem. 2016, 8, 692–697. 10.1038/nchem.2524. 27325096

[ref9] Lu Z.; Beaulieu L. Y.; Donaberger R. A.; Thomas C. L.; Dahn J. R. Synthesis, Structure, and Electrochemical Behavior of Li[Ni_x_Li_1/3–2x/3_Mn_2/3–x/3_]O_2_. J. Electrochem. Soc. 2002, 149, A778 10.1149/1.1471541.

[ref10] Lu Z.; Dahn J. R. Understanding the Anomalous Capacity of Li/Li[Ni_x_Li_(1/3–2x/3)_Mn_(2/3–x/3)_]O_2_ Cells Using In Situ X-Ray Diffraction and Electrochemical Studies. J. Electrochem. Soc. 2002, 149, A815 10.1149/1.1480014.

[ref11] House R. A.; Maitra U.; Perez-Osorio M. A.; Lozano J. G.; Jin L.; Somerville J. W.; Duda L. C.; Nag A.; Walters A.; Zhou K. J.; Roberts M. R.; Bruce P. G. Superstructure control of first-cycle voltage hysteresis in oxygen-redox cathodes. Nature 2020, 577, 502–508. 10.1038/s41586-019-1854-3. 31816625

[ref12] Sathiya M.; Rousse G.; Ramesha K.; Laisa C. P.; Vezin H.; Sougrati M. T.; Doublet M. L.; Foix D.; Gonbeau D.; Walker W.; Prakash A. S.; Ben Hassine M.; Dupont L.; Tarascon J. M. Reversible anionic redox chemistry in high-capacity layered-oxide electrodes. Nat. Mater. 2013, 12, 827–835. 10.1038/nmat3699. 23852398

[ref13] Ben Yahia M.; Vergnet J.; Saubanere M.; Doublet M. L. Unified picture of anionic redox in Li/Na-ion batteries. Nat. Mater. 2019, 18, 496–502. 10.1038/s41563-019-0318-3. 30886397

[ref14] Hong J.; Gent W. E.; Xiao P.; Lim K.; Seo D.-H.; Wu J.; Csernica P. M.; Takacs C. J.; Nordlund D.; Sun C.-J.; Stone K. H.; Passarello D.; Yang W.; Prendergast D.; Ceder G.; Toney M. F.; Chueh W. C. Metal–oxygen decoordination stabilizes anion redox in Li-rich oxides. Nat. Mater. 2019, 18, 256–265. 10.1038/s41563-018-0276-1. 30718861

[ref15] House R. A.; Rees G. J.; Pérez-Osorio M. A.; Marie J.-J.; Boivin E.; Robertson A. W.; Nag A.; Garcia-Fernandez M.; Zhou K.-J.; Bruce P. G. First-cycle voltage hysteresis in Li-rich 3d cathodes associated with molecular O_2_ trapped in the bulk. Nat. Energy 2020, 5, 777–785. 10.1038/s41560-020-00697-2.

[ref16] Fell C. R.; Carroll K. J.; Chi M.; Meng Y. S. Synthesis–Structure–Property Relations in Layered, “Li-excess” Oxides Electrode Materials Li[Li_1 / 3 – 2x / 3_Ni_x_Mn_2 / 3 – x / 3_ ] O_2_ (x = 1 / 3 , 1/4, and 1/5). J. Electrochem. Soc. 2010, 157 (57), A1202 10.1149/1.3473830.

[ref17] Li B.; Kumar K.; Roy I.; Morozov A. V.; Emelyanova O. V.; Zhang L.; Koç T.; Belin S.; Cabana J.; Dedryvère R.; et al. Capturing dynamic ligand-to-metal charge transfer with a long-lived cationic intermediate for anionic redox. Nat. Mater. 2022, 21, 1165–1174. 10.1038/s41563-022-01278-2. 35725928

[ref18] Grey C. P.; Yoon W.-S.; Reed J.; Ceder G. Electrochemical Activity of Li in the Transition-Metal Sites of O3 Li[Li_( 1 – 2x ) /3_Mn_( 2 – x ) /3_Ni_x_]O_2_. Electrochem. Solid-State Lett. 2004, 7, A290 10.1149/1.1783113.

[ref19] Xu B.; Fell C. R.; Chi M.; Meng Y. S. Identifying surface structural changes in layered Li-excess nickel manganese oxides in high voltage lithium ion batteries: A joint experimental and theoretical study. Energy Environ. Sci. 2011, 4, 2223 10.1039/c1ee01131f.

[ref20] Ku K.; Kim B.; Jung S.-K.; Gong Y.; Eum D.; Yoon G.; Park K.-Y.; Hong J.; Cho S.-P.; Kim D.-H.; Kim H.; Jeong E.; Gu L.; Kang K. A new lithium diffusion model in layered oxides based on asymmetric but reversible transition metal migration. Energy Environ. Sci. 2020, 13, 1269–1278. 10.1039/C9EE04123K.

[ref21] Song W. X.; Perez-Osorio M. A.; Marie J. J.; Liberti E.; Luo X. N.; O’Leary C.; House R. A.; Bruce P. G.; Nellist P. D. Direct imaging of oxygen shifts associated with the oxygen redox of Li-rich layered oxides. Joule 2022, 6, 1049–1065. 10.1016/j.joule.2022.04.008.

[ref22] Hao B.; Ding Z.; Tao X.; Nellist P. D.; Assender H. E. Atomic-scale imaging of polyvinyl alcohol crystallinity using electron ptychography. Polymer 2023, 284, 126305 10.1016/j.polymer.2023.126305.

[ref23] Clark L.; Martinez G. T.; O’Leary C. M.; Yang H.; Ding Z.; Petersen T. C.; Findlay S. D.; Nellist P. D. The Effect of Dynamical Scattering on Single-plane Phase Retrieval in Electron Ptychography. Microsc. Microanal. 2023, 29, 384–394. 10.1093/micmic/ozac022.37613463

[ref24] Yang H.; Rutte R. N.; Jones L.; Simson M.; Sagawa R.; Ryll H.; Huth M.; Pennycook T. J.; Green M. L. H.; Soltau H.; et al. Simultaneous atomic-resolution electron ptychography and Z-contrast imaging of light and heavy elements in complex nanostructures. Nat. Commun. 2016, 7, 12532 10.1038/ncomms12532. 27561914 PMC5007440

[ref25] Chen Z.; Jiang Y.; Shao Y. T.; Holtz M. E.; Odstrcil M.; Guizar-Sicairos M.; Hanke I.; Ganschow S.; Schlom D. G.; Muller D. A. Electron ptychography achieves atomic-resolution limits set by lattice vibrations. Science 2021, 372, 826–831. 10.1126/science.abg2533. 34016774

[ref26] Chen J.; Zhou J.; Xu W.; Wen Y.; Liu Y.; Warner J. H. Atomic-Level Dynamics of Point Vacancies and the Induced Stretched Defects in 2D Monolayer PtSe_2_. Nano Lett. 2022, 22, 3289–3297. 10.1021/acs.nanolett.1c04275. 35389659

[ref27] Shukla A. K.; Ramasse Q. M.; Ophus C.; Duncan H.; Hage F.; Chen G. Unravelling structural ambiguities in lithium- and manganese-rich transition metal oxides. Nat. Commun. 2015, 6, 8711 10.1038/ncomms9711. 26510508 PMC4846316

[ref28] Yang H.; MacLaren I.; Jones L.; Martinez G. T.; Simson M.; Huth M.; Ryll H.; Soltau H.; Sagawa R.; Kondo Y.; Ophus C.; Ercius P.; Jin L.; Kovács A.; Nellist P. D. Electron ptychographic phase imaging of light elements in crystalline materials using Wigner distribution deconvolution. Ultramicroscopy 2017, 180, 173–179. 10.1016/j.ultramic.2017.02.006. 28434783

[ref29] Zhao E.; Zhang M.; Wang X.; Hu E.; Liu J.; Yu X.; Olguin M.; Wynn T. A.; Meng Y. S.; Page K.; Wang F.; Li H.; Yang X.-Q.; Huang X. Local structure adaptability through multi cations for oxygen redox accommodation in Li-Rich layered oxides. Energy Storage Mater. 2020, 24, 384–393. 10.1016/j.ensm.2019.07.032.

[ref30] Bréger J.; Meng Y. S.; Hinuma Y.; Kumar S.; Kang K.; Shao-Horn Y.; Ceder G.; Grey C. P. Effect of High Voltage on the Structure and Electrochemistry of LiNi_0.5_Mn_0.5_O_2_: A Joint Experimental and Theoretical Study. Chem. Mater. 2006, 18, 4768–4781. 10.1021/cm060886r.

[ref31] Wang C.; Wang X.; Zhang R.; Lei T.; Kisslinger K.; Xin H. L. Resolving complex intralayer transition motifs in high-Ni-content layered cathode materials for lithium-ion batteries. Nat. Mater. 2023, 22, 235–241. 10.1038/s41563-022-01461-5. 36702885

[ref32] House R. A.; Playford H. Y.; Smith R. I.; Holter J.; Griffiths I.; Zhou K.-J.; Bruce P. G. Detection of trapped molecular O_2_ in a charged Li-rich cathode by Neutron PDF. Energy Environ. Sci. 2022, 15, 376–383. 10.1039/D1EE02237G.

[ref33] Cui C.; Fan X.; Zhou X.; Chen J.; Wang Q.; Ma L.; Yang C.; Hu E.; Yang X.-Q.; Wang C. Structure and Interface Design Enable Stable Li-Rich Cathode. J. Am. Chem. Soc. 2020, 142, 8918–8927. 10.1021/jacs.0c02302. 32319764

[ref34] Yang Z.; Zhong J.; Liu Y.; Li Z.; Li J.; Yang K. Unveiling the Effect of Voltage Regulation System on the Structure and Electrochemical Properties of Lithium-Rich Cathode Materials. J. Electrochem. Soc. 2019, 166, A1481–A1489. 10.1149/2.0371908jes.

[ref35] Yan P.; Nie A.; Zheng J.; Zhou Y.; Lu D.; Zhang X.; Xu R.; Belharouak I.; Zu X.; Xiao J.; Amine K.; Liu J.; Gao F. Evolution of Lattice Structure and Chemical Composition of the Surface Reconstruction Layer in Li_1.2_Ni_0.2_Mn_0.6_O_2_ Cathode Material for Lithium Ion Batteries. Nano Lett. 2015, 15, 514–522. 10.1021/nl5038598. 25485638

[ref36] Van der Ven A.; Bhattacharya J.; Belak A. A. Understanding Li Diffusion in Li-Intercalation Compounds. Acc. Chem. Res. 2013, 46, 1216–1225. 10.1021/ar200329r. 22584006

[ref37] Zeng Y.; Ouyang B.; Liu J.; Byeon Y. W.; Cai Z.; Miara L. J.; Wang Y.; Ceder G. High-entropy mechanism to boost ionic conductivity. Science 2022, 378, 1320–1324. 10.1126/science.abq1346. 36548421

[ref38] Zheng J.; Ye Y.; Liu T.; Xiao Y.; Wang C.; Wang F.; Pan F. Ni/Li Disordering in Layered Transition Metal Oxide: Electrochemical Impact, Origin, and Control. Acc. Chem. Res. 2019, 52, 2201–2209. 10.1021/acs.accounts.9b00033. 31180201

[ref39] Giannozzi P.; Baroni S.; Bonini N.; Calandra M.; Car R.; Cavazzoni C.; Ceresoli D.; Chiarotti G. L.; Cococcioni M.; Dabo I.; Dal Corso A.; de Gironcoli S.; Fabris S.; Fratesi G.; Gebauer R.; Gerstmann U.; Gougoussis C.; Kokalj A.; Lazzeri M.; Martin-Samos L.; Marzari N.; Mauri F.; Mazzarello R.; Paolini S.; Pasquarello A.; Paulatto L.; Sbraccia C.; Scandolo S.; Sclauzero G.; Seitsonen A. P.; Smogunov A.; Umari P.; Wentzcovitch R. M. QUANTUM ESPRESSO: A modular and open-source software project for quantum simulations of materials. J. Phys.: Condens. Matter 2009, 21, 395502 10.1088/0953-8984/21/39/395502. 21832390

[ref40] Cococcioni M.; de Gironcoli S. Linear response approach to the calculation of the effective interaction parameters in the LDA+U method. Phys. Rev. B 2005, 71, 035105 10.1103/PhysRevB.71.035105.

[ref41] Perdew J. P.; Burke K.; Ernzerhof M. Generalized Gradient Approximation Made Simple. Phys. Rev. Lett. 1996, 77, 3865–3868. 10.1103/PhysRevLett.77.3865. 10062328

[ref42] Heyd J.; Scuseria G. E.; Ernzerhof M. Hybrid functionals based on a screened Coulomb potential. J. Chem. Phys. 2003, 118, 8207–8215. 10.1063/1.1564060.

[ref43] Kang S.; Mo Y.; Ong S. P.; Ceder G. A Facile Mechanism for Recharging Li_2_O_2_ in Li–O_2_ Batteries. Chem. Mater. 2013, 25, 3328–3336. 10.1021/cm401720n.

