# Supplementary material for: Visualization of Tetrahedral Li in the Alkali Layers of Li-Rich Layered Metal Oxides
Source: J Am Chem Soc. 2024 Aug 14;146(34):23814–24. doi: 10.1021/jacs.4c05556 (PMC11363138; doi:10.1021/jacs.4c05556)
Supplement: Supplementary file 1 — ja4c05556_si_001.pdf [file ja4c05556_si_001.pdf]

Supplementary Information

Visualisation of tetrahedral Li in the alkali layers of Li-rich layered metal oxides

Weixin Song[1,2,3]*, Miguel A. Pérez-Osorio[1,2,3], Jun Chen[1,3], Zhiyuan Ding[1,3], John-Joseph Marie[1,2,3], Mikkel Juelsholt[1,2,3], Robert A. House[1,2,3], Peter G. Bruce[1,2,3], Peter D. Nellist[1,2,3]*

[1]Department of Materials, University of Oxford, Oxford OX1 3PH, UK

[2]The Faraday Institution, Didcot OX11 0RA, UK

[3] The Henry Royce Institute, Oxford OX1 3PH, UK

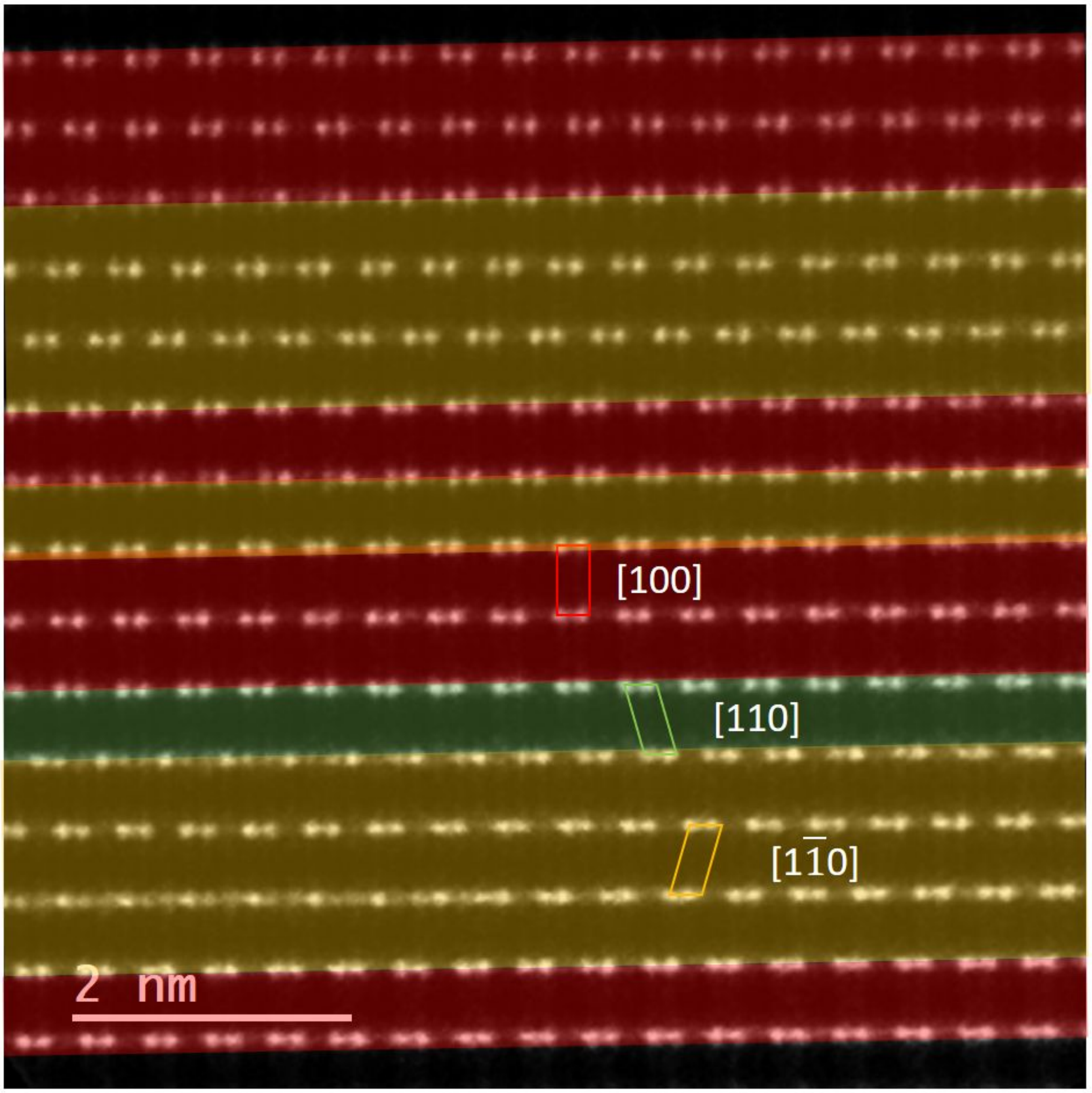

Figure S1 ADF image of pristine $Li_{1.2}Ni_{0.13}Mn_{0.54}Co_{0.13}O_2$ along the mixed zone axis of [100], [110] or [1$\bar{1}$0]. The various domains visible are consistent with projection along the [100], [110] and [1$\bar{1}$0] zone axis of a crystal of the *C2/m* space group as indicated.

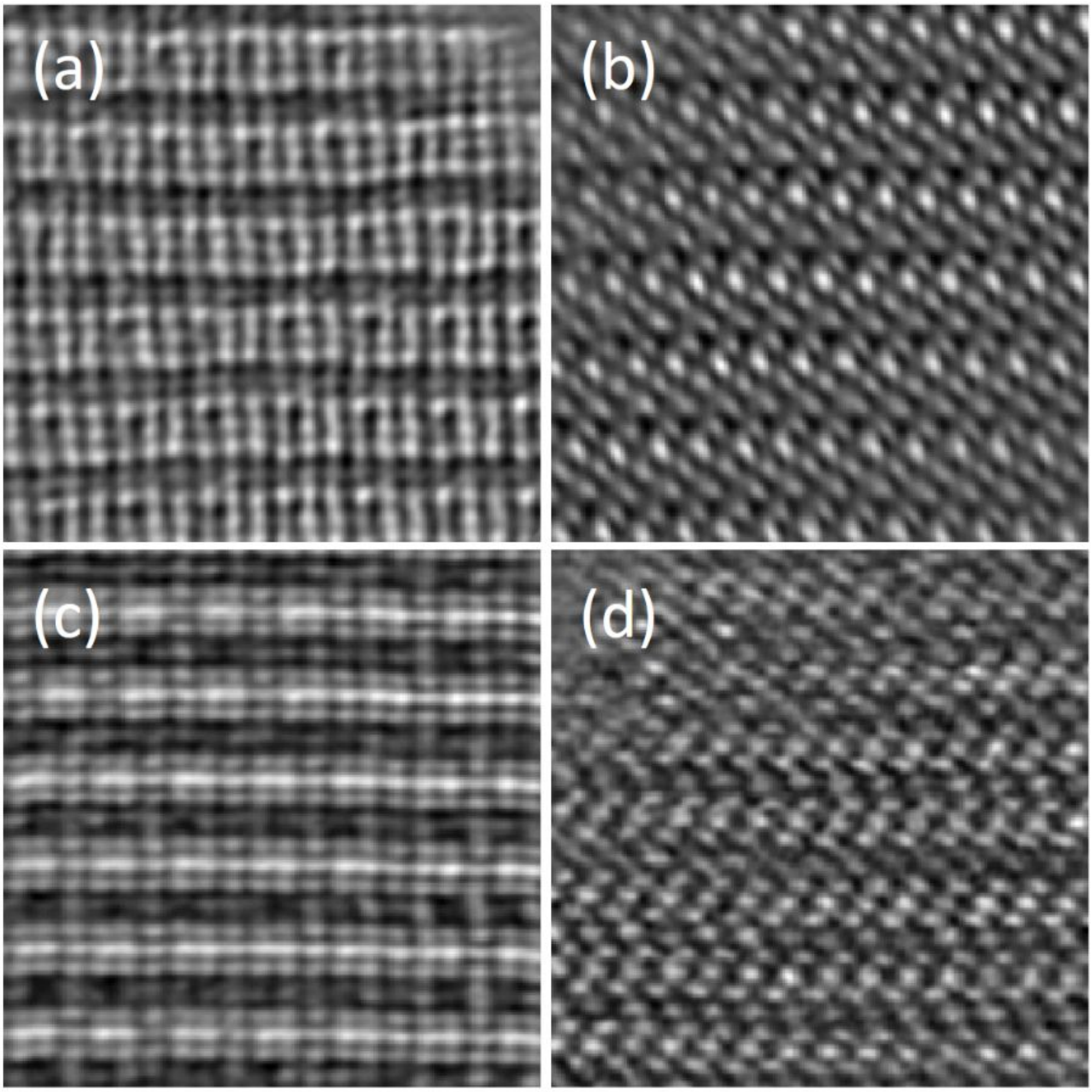

Figure S2 Ptychographic phase images of $Li_{1.2}Ni_{0.13}Mn_{0.54}Co_{0.13}O_2$. (a, b) Pristine state. (c, d) BOP state.

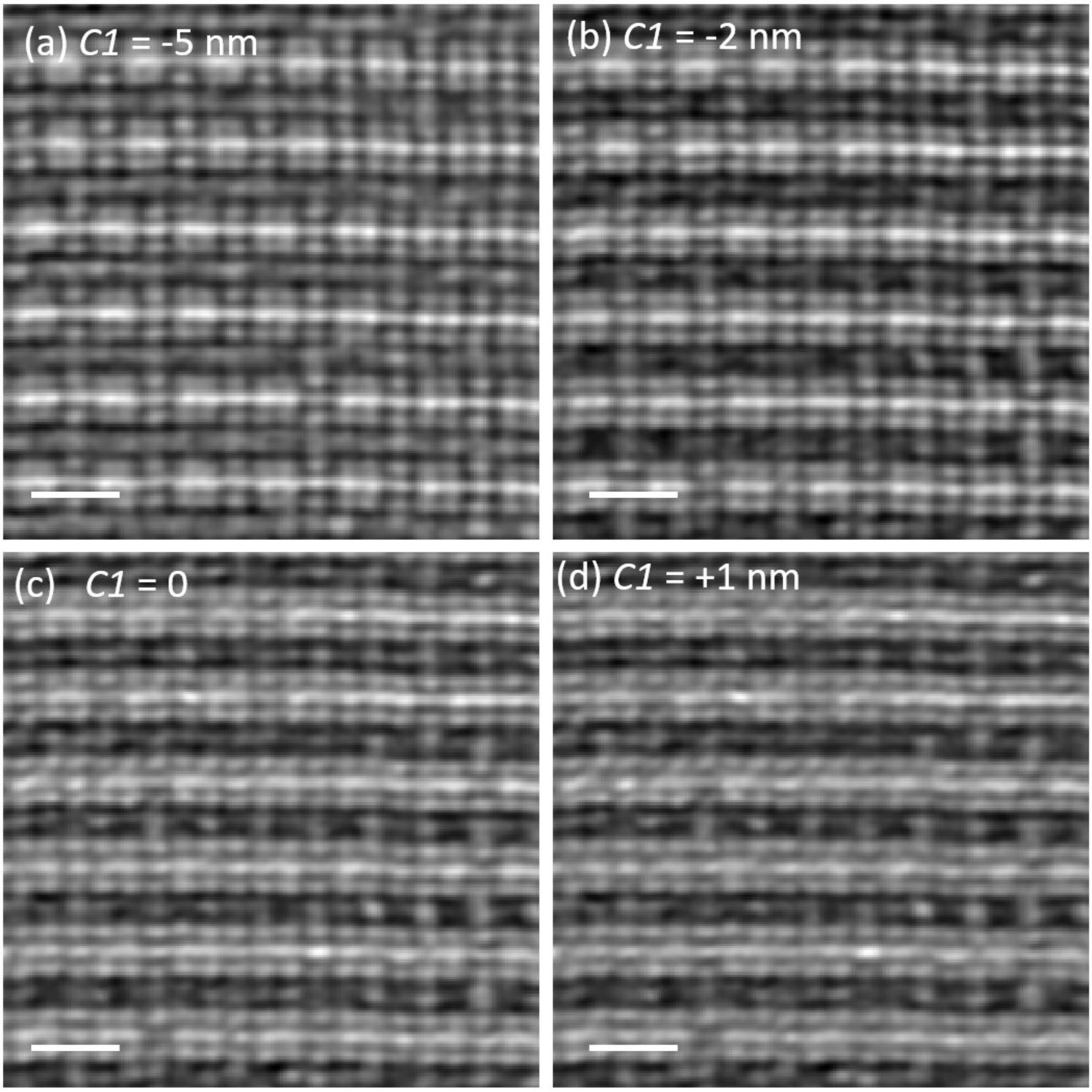

Figure S3 WDD optical sectioning of $Li_{1.2}Ni_{0.13}Mn_{0.54}Co_{0.13}O_2$ of BOP state. '-' means under focus relative to the experimental focal plane. The phase image at *C1* = -2 nm shows better visible contrast than the other focal planes and is considered as a bulk-representitive image with minimal surface effect.

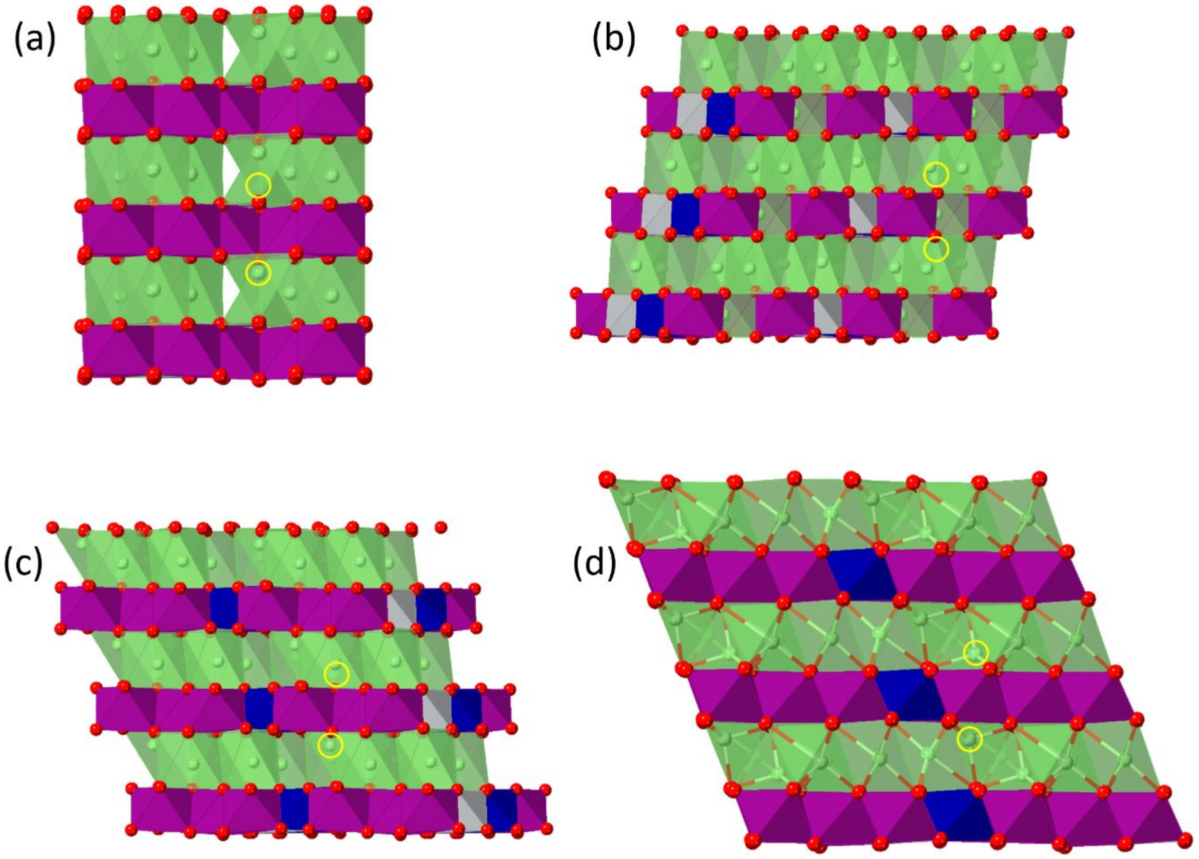

Figure S4 Schematic representation of the tetrahedral Li-Li dumbbells in BOP $Li_{1.2}Ni_{0.13}Mn_{0.54}Co_{0.13}O_2$. (a) [100] zone axis. (b) [$1\overline{1}0$] zone axis. (c) [110] zone axis. (d) [010] zone axis. The superimposed yellow circles are used to label a tetrahedral Li-Li dumbbell as an example. The purple represents Mn, blue Co, grey Ni, red O and green Li.

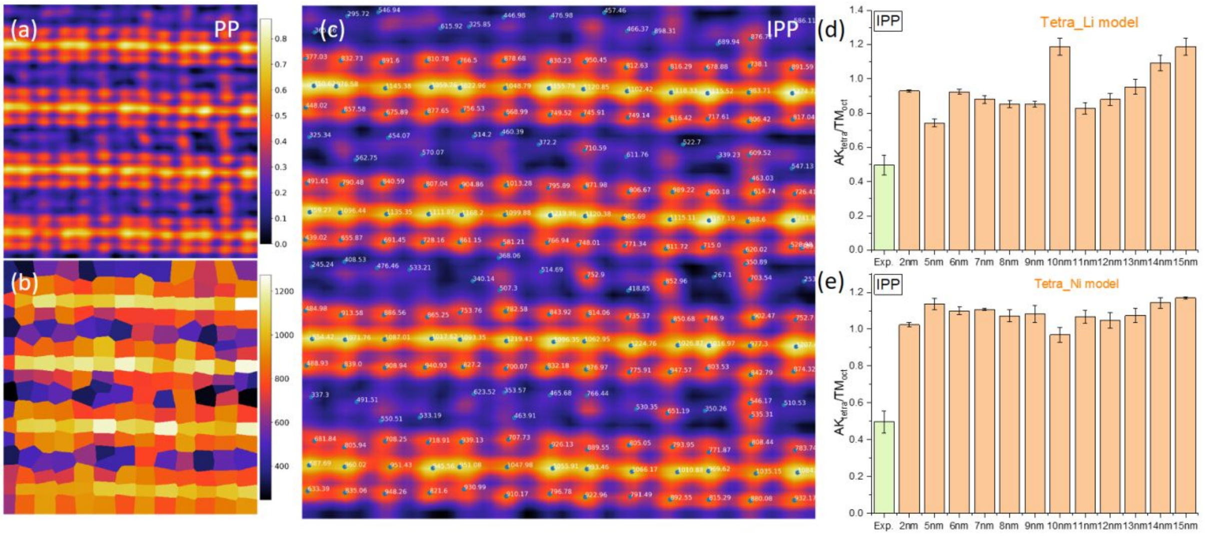

Figure S5 Mathematical processing of the ptychographic phase by lifting the phase contrast minimum to be 0. (a) The formed positive phase (PP) image. (b) Diagram of Voronoi cells. (c) The integral PP values over each atom in the PP image. (d) Plots of the integral PP ratios from the reconstructed ptychographic image of the sample and the simulated image of the tetrahedral Li model. (e) Plots of the integral PP ratios from the reconstructed ptychographic image of the sample and the simulated image of the tetrahedral Ni model.

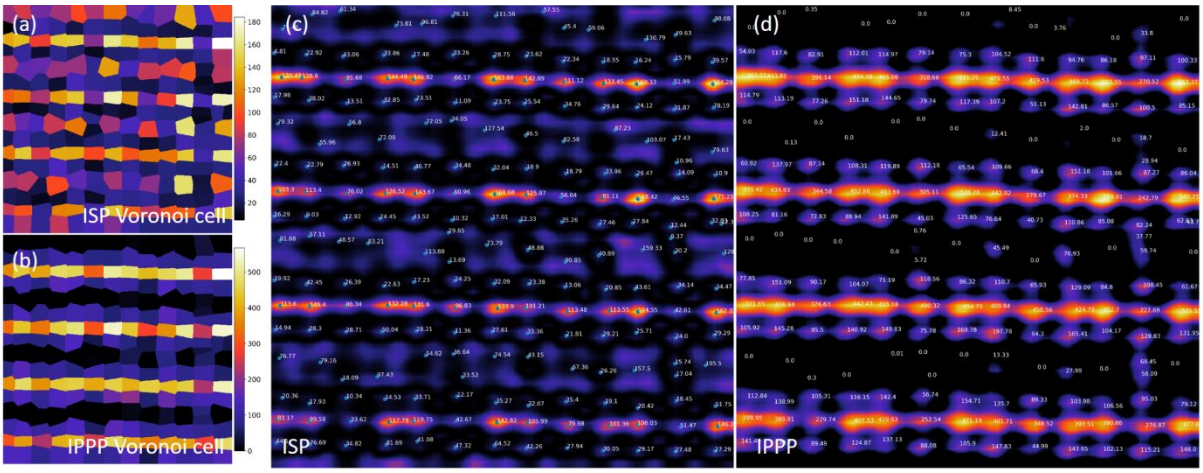

Figure S6 Integration of the values in SP and PPP images. Diagram of the used Voronoi cells in (a) SP and (b) PPP image. (c) Integral SP values over the atoms displayed in the SP image. (d) Integral PPP values over the atoms displayed in the PPP image.

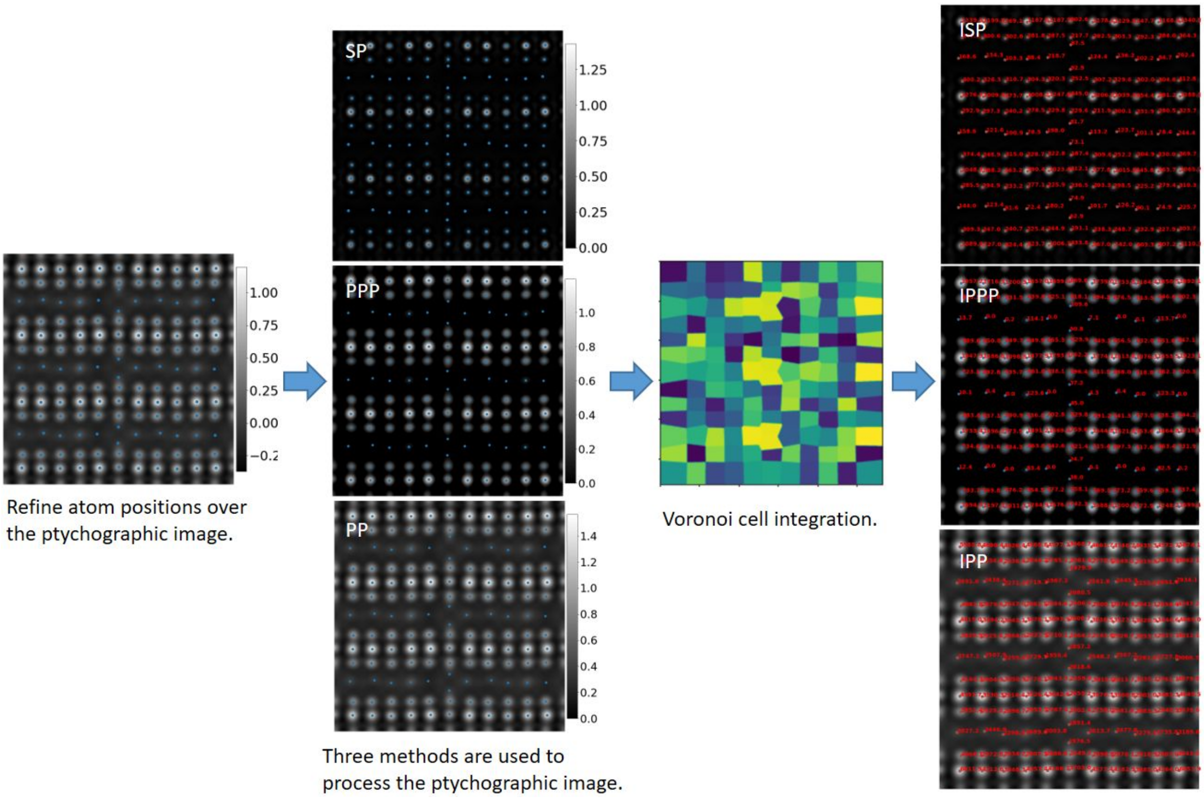

Figure S7 Protocols for quantitative use of the ptychographic phase. The first step is to define the atom positions over the reconstructed ptychographic image using Atomap software. The ptychographic image is simulated using the BOP LRNMC model with a 5 nm thickness. The second step is to process the ptychographic phase image using three mathematic methods and produce the SP, PPP and PP images. The third step is to implement Voronoi cells around each atom position and integrate the SP, PPP and PP values to result in the integral quantities. The detailed values can be seen clearly from the following SI figures.

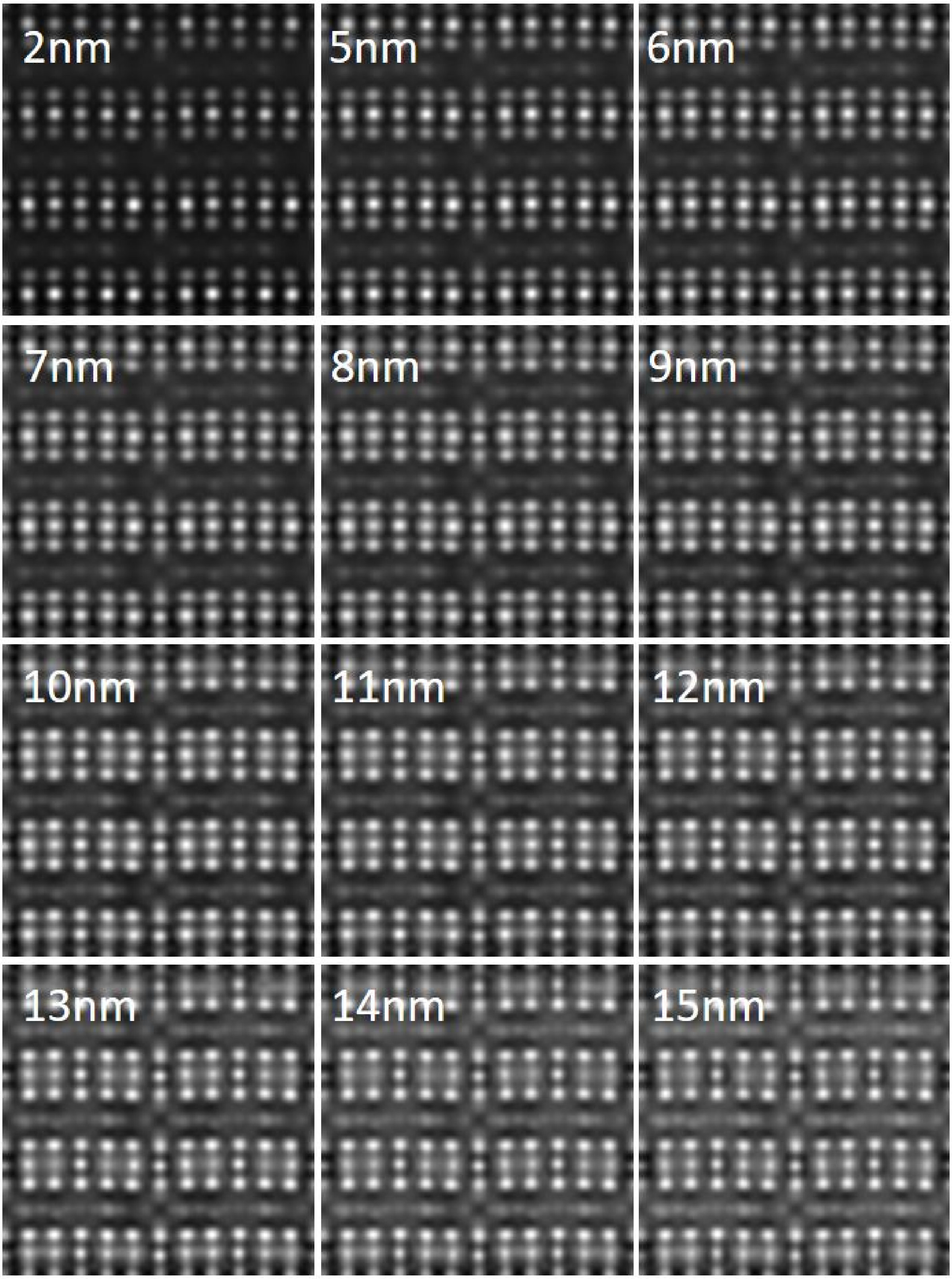

Figure S8 Simulated ptychographic images of tetrahedral Li model with a number of thicknesses from 2nm to 15 nm.

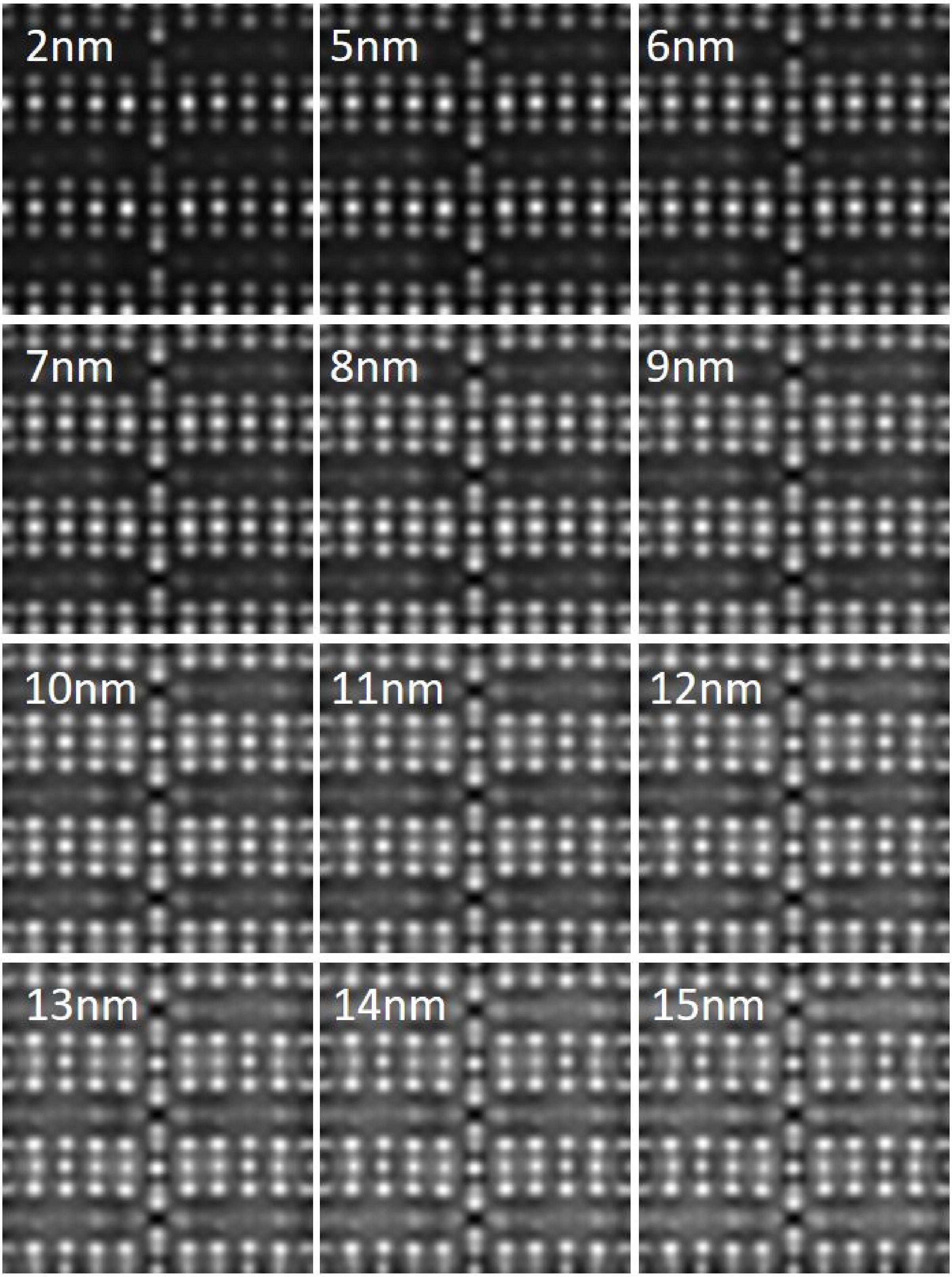

Figure S9 Simulated ptychographic images of tetrahedral Ni model with a number of thicknesses from 2nm to 15 nm.

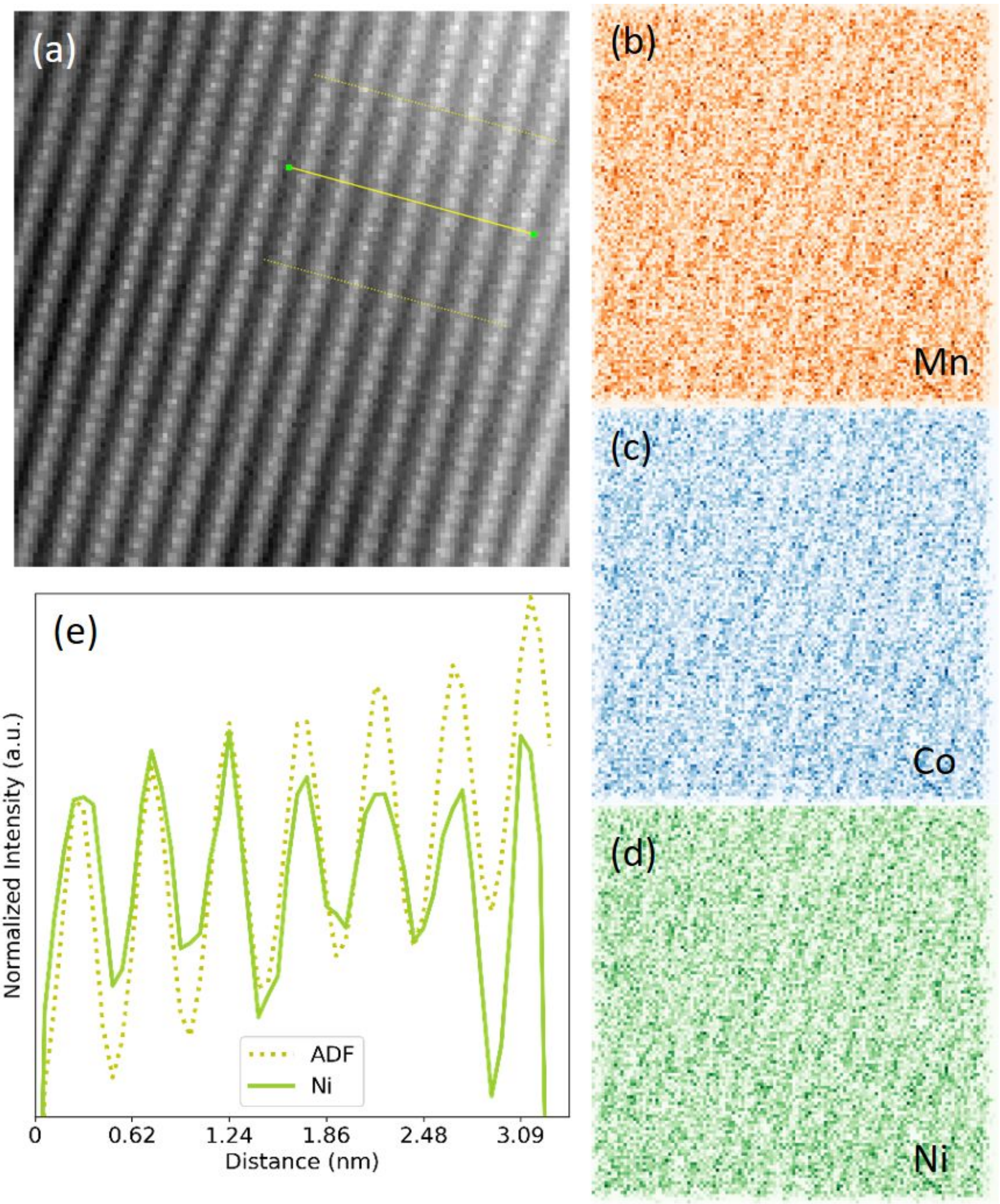

Figure S10 EDX spectroscopy of BOP $Li_{1.2}Ni_{0.13}Mn_{0.54}Co_{0.13}O_2$. (a-c) Atomic resolution EDX mapping of Mn, Co and Ni respectively. (d) ADF image where EDX mappings are collected. The yellow lines label the line profile region which is also applied to the Ni mapping image. (e) Line profiles of the Ni mapping and ADF image. The multi-frame EDX mappings are aligned using Hyperspy align2D function using phase correlation algorithems.

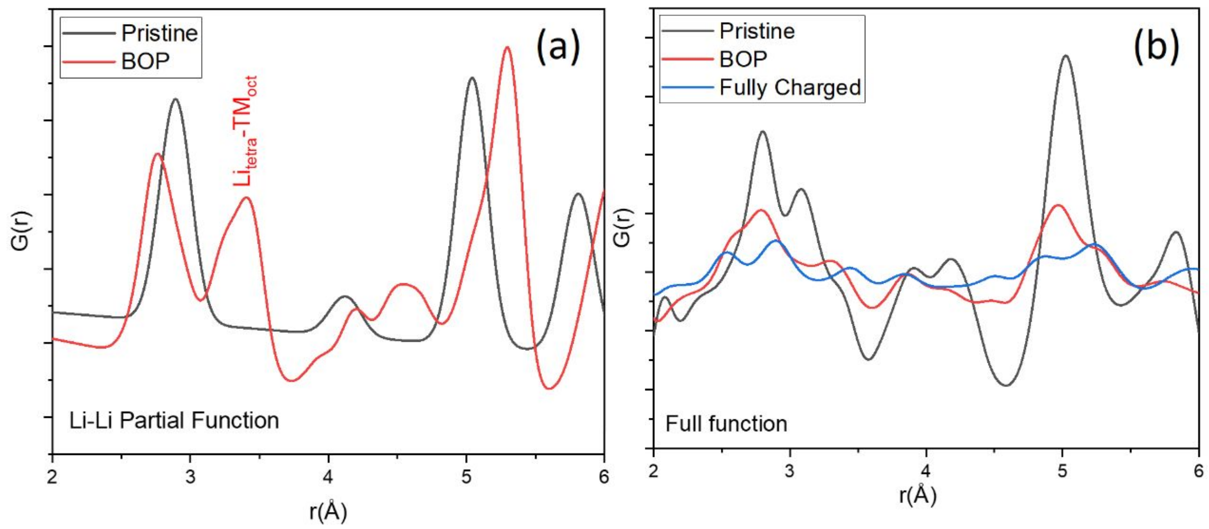

Figure S11 Simulated neutron PDF of LRNMC at pristine, BOP and FC state using the models from DFT. (a) Li-Li partial function. (b) Full function.

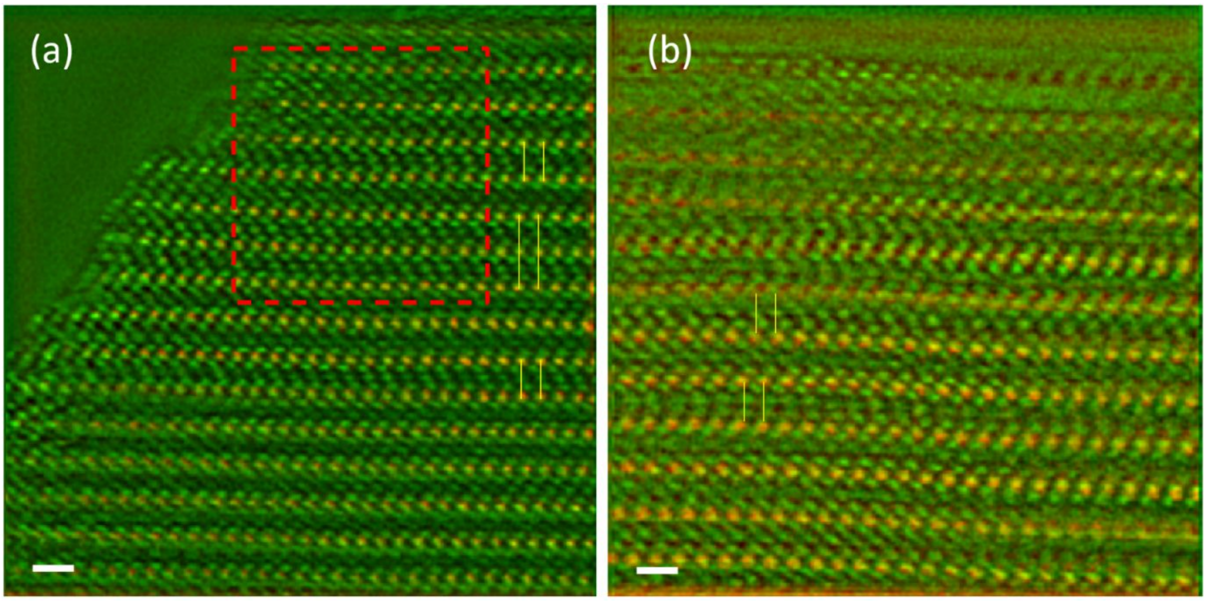

Figure S12 Coloured ptychographic phase image of BOP LRNMC from two local areas. Both images have a field of view of ~7.2x7.2 nm. The yellow vertical lines indicate the layers showing the O1 phase. The red square in (a) represents the area displayed in Figure 1l. The scale bar is 0.5 nm.

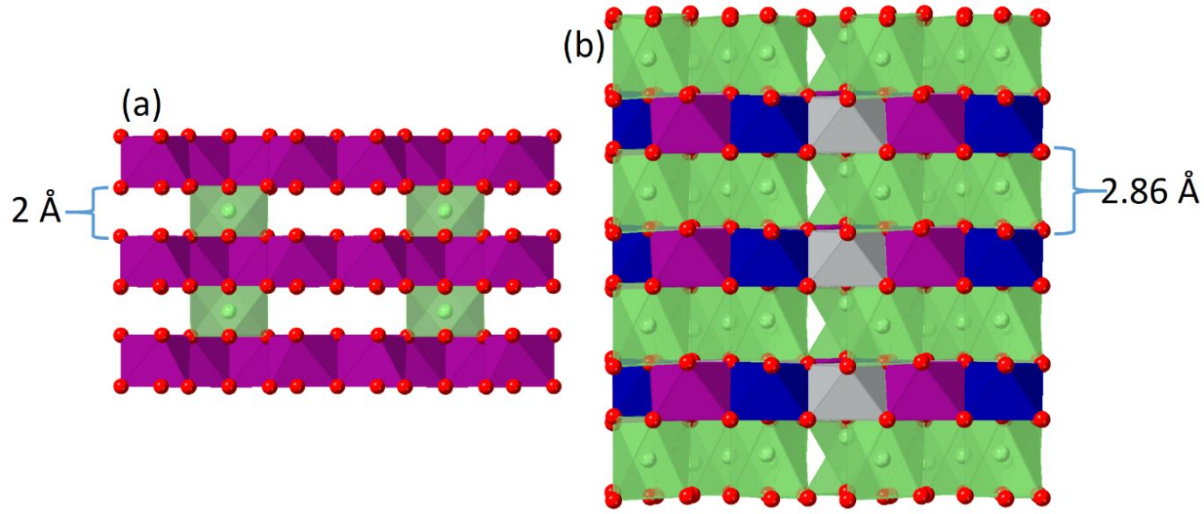

Figure S13 DFT calculations of the O-O layer spacing in the alkali layers of the O3 and O1 phases at the BOP state. (a) O1 phase. (b) O3 phase.

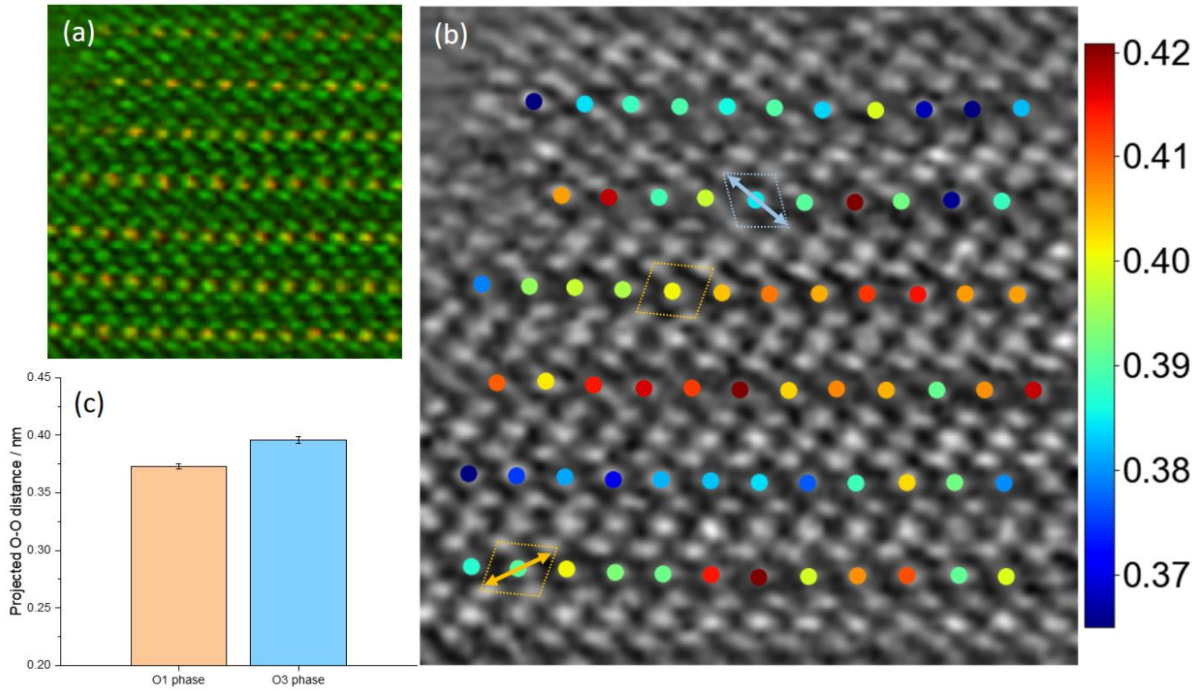

Figure S14 Measurement of the projected O-O length along the axial direction of the alkali-layer octahedra. The axial direction of the O1-phase octahedra is shown in blue and the O3-phase in orange, (a) Coloured ptychographic image of BOP LRNMC. (b) Projected O-O lengths over the ptychographic image. (c) The average of the projected O-O lengths and standard errors from the O1 and O3-phase regions, respectively.

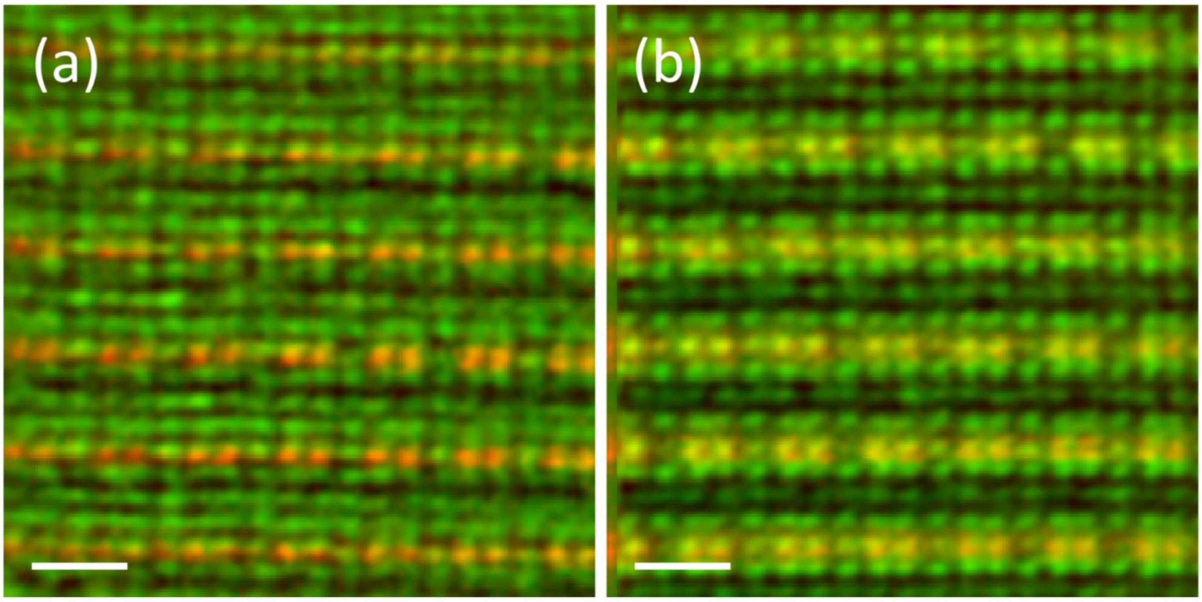

Figure S14 Coloured ptychographic images of FD LRNMC from different cut-offs. (a) 4.8 V. (b) 4.45 V.
